# Recent therapeutic advances in gynecologic oncology: evolving roles of immunotherapy, antibody–drug conjugates, and clinical trial innovations

**DOI:** 10.3389/fonc.2025.1697180

**Published:** 2026-01-15

**Authors:** Gaukhar Koshkimbayeva, Akerke Amirkhanova, Aiken Orazymbetova, Alma Nurakhova, Akmaral Maimakova, Altyn Duisenbayeva, Nurgulim Akhmad, Altyn Abilova, Arailym Abilbayeva, Sholpan Akhelova, Dana Akhmentayeva, Aida Seitaliyeva, Zaure Dushimova, Zhanserik Shynykul, Sandugash Yerkenova

**Affiliations:** 1 Department of General Medical Practice with Courses, Kazakh-Russian Medical University, Almaty, Kazakhstan; 2 School of Pharmacy, S.D. Asfendiyarov Kazakh National Medical University, Almaty, Kazakhstan; 3 Department of Anatomy with Courses, Kazakh-Russian Medical University, Almaty, Kazakhstan; 4 Department of Normal Physiology with a course in Biophysics, School of Medicine, S.D. Asfendiyarov Kazakh National Medical University, Almaty, Kazakhstan; 5 Clinical Medicine Department of International Business, University named after Kenzhegali Sagadiev, Almaty, Kazakhstan; 6 Department of General Medical Practice No. 2, School of Medicine, S.D. Asfendiyarov Kazakh National Medical University, Almaty, Kazakhstan; 7 Department of Anatomy named after S. R. Karynbayev, School of General Medicine 1, S.D. Asfendiyarov Kazakh National Medical University, Almaty, Kazakhstan; 8 General Immunology Department, S.D. Asfendiyarov Kazakh National Medical University, Almaty, Kazakhstan; 9 Department of Pharmaceutical Disciplines, NCJSC “Astana Medical University”, Astana, Kazakhstan; 10 Department of Internal Medicine, S.D. Asfendiyarov Kazakh National Medical University, Almaty, Kazakhstan; 11 Faculty of Medicine and Healthcare, Al-Farabi Kazakh National University, Almaty, Kazakhstan; 12 Department of Anatomy, Kazakhstan’s Medical University “KSPH”, Almaty, Kazakhstan

**Keywords:** gynecologic malignancies, immune checkpoint inhibitors, antibody–drug conjugates, ovarian cancer, endometrial cancer, cervical cancer, cancer

## Abstract

**Background and objectives:**

Gynecologic cancers, including cervical, endometrial, and ovarian malignancies, remain among the leading causes of cancer-related illness and death in women worldwide. Despite progress in surgery and chemotherapy, resistance to conventional cytotoxic drugs continues to limit durable outcomes. The introduction of immune checkpoint inhibitors (ICIs) and antibody–drug conjugates (ADCs) has created new therapeutic opportunities by improving survival and overcoming resistance mechanisms. This review summarizes the latest clinical evidence on immunotherapy and ADC-based regimens, emphasizing their integration into current treatment strategies and the expanding roles of genomic profiling and artificial intelligence (AI) in personalized therapy.

**Materials and methods:**

Recent findings from major clinical trials such as RUBY, NRG-GY018, DUO-O, SORAYA, and DESTINY-PanTumor02 were evaluated along with updated FDA and NCCN recommendations. The analysis focuses on treatments that have demonstrated clinical benefit in advanced or recurrent disease, including pembrolizumab, dostarlimab, tisotumab vedotin, and mirvetuximab soravtansine. Combination strategies incorporating PARP inhibitors, antiangiogenic agents, and immune checkpoint blockade were also reviewed.

**Results:**

Checkpoint inhibitors have achieved meaningful clinical benefits in patients with advanced or recurrent endometrial and cervical cancers, particularly in those with mismatch repair deficiency or PD-L1 expression. ADCs directed against tissue factor (TF) and folate receptor alpha have shown effectiveness in platinum-resistant cervical and ovarian cancers. Combination regimens that include ICIs, PARP inhibitors, or antiangiogenic therapy are yielding encouraging results in both first-line and maintenance settings. Advances in molecular profiling and biomarker-based patient selection, supported by AI applications, are further improving treatment precision in gynecologic oncology.

**Conclusions:**

Immunotherapy and ADCs represent major advances in the treatment of gynecologic cancers. Their growing integration into clinical practice has reshaped therapeutic approaches, while ongoing research continues to refine optimal combinations, address resistance, and enhance biomarker-guided selection. Future developments are expected to unite immunologic, genomic, and computational strategies to achieve personalized and durable outcomes for patients with gynecologic malignancies.

## 1 Introduction

The occurrence of gynecologic cancers impacting the female reproductive system has been steadily rising, primarily due to modern lifestyle habits, unhealthy diets, and genetic predispositions (1). These cancers encompass vulvar, uterine, vaginal, cervical, ovarian (OC), and fallopian-tube tumors, each classified according to anatomical origin. Fallopian-tube carcinoma remains exceptionally uncommon, accounting for less than 1% of female genital-tract malignancies (2, 3). In contrast, endometrial, ovarian, and cervical cancers are the most prevalent, together representing about 35–40% of cancers diagnosed in women worldwide (4). Globally, cervical cancer ranks fourth among cancers in women, with approximately 604–000 new cases and 342–000 deaths in 2020, most occurring in low- and middle-income countries (5). Endometrial (uterine) cancer is the sixth most common, causing roughly 417–000 cases and 97–000 deaths annually (6). Ovarian cancer, often detected at advanced stages, ranks eighth, with 314–000 new cases and 207–000 deaths per year (7). Variations in incidence and mortality reflect access to screening, healthcare quality, reproductive behavior, and socioeconomic status (8). These patterns underscore the global need for improved prevention, early detection, and individualized treatment strategies.

The treatment of gynecologic cancers remains challenging, especially in advanced or recurrent disease (9). Historically, carboplatin and paclitaxel have been the backbone of systemic therapy, acting through DNA cross-linking and microtubule stabilization, respectively (10). However, resistance often develops due to enhanced DNA repair, survival-pathway activation, and altered drug metabolism (11). Advances in molecular oncology and immunotherapy have revolutionized treatment paradigms by targeting tumor-specific mutations and immune-escape mechanisms. Agents such as bevacizumab (anti-angiogenic), PARP inhibitors (e.g., olaparib), and immune checkpoint inhibitors directed at PD-1/PD-L1 have become pivotal (12, 13). Bevacizumab received FDA approval in 2014 for ovarian cancer in platinum-resistant settings, while olaparib was first approved in 2014 for BRCA-mutated OC. Checkpoint inhibitors such as pembrolizumab were approved in 2017 for MSI-H or mismatch repair deficient (dMMR) solid tumors, including endometrial cancer. These therapies exemplify precision medicine by aligning treatment selection with molecular and immune biomarkers (14).

**Figure 1:**
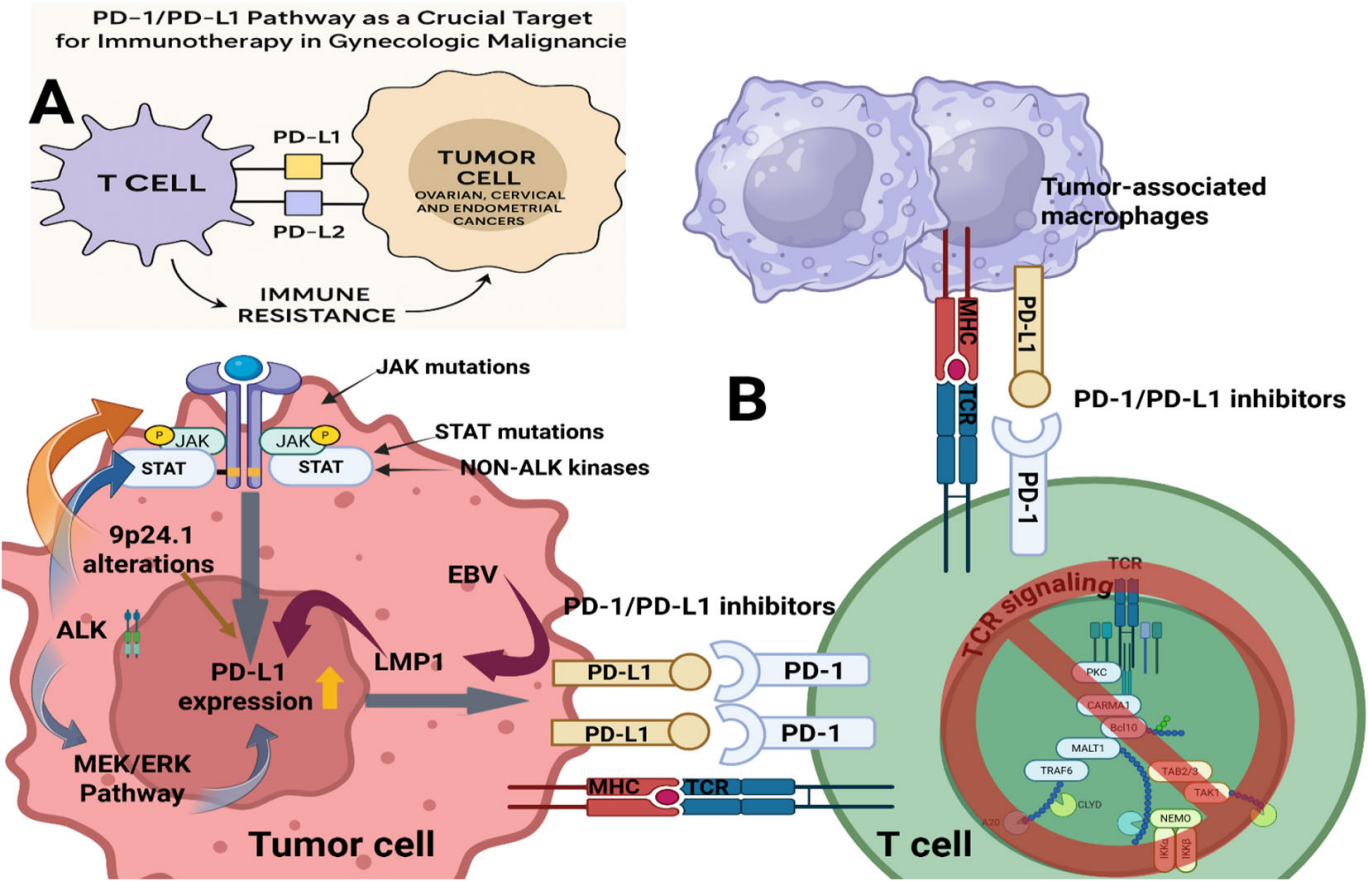
Mechanisms of PD-1/PD-L1-mediated immune evasion and therapeutic blockade in gynecologic malignancies: **(A)** The interplay between tumor cells and T-cells via PD-1/PD-L1 and PD-1/PD-L2 pathways in ovarian, cervical, and endometrial tumors. **(B)** Immune suppression involves tumor cells, tumor-associated macrophages (TAMs), and T-cells. Upregulation of PD-L1 occurs through 9p24.1 amplifications, JAK/STAT activation, kinase mutations, EBV LMP1, and MEK/ERK signaling. Therapeutic antibodies interrupt these inhibitory interactions, restoring T-cell cytotoxicity.

Immune evasion constitutes a central obstacle to durable therapeutic response. The PD-1 receptor and its ligands PD-L1 and PD-L2 act as immune-checkpoint regulators that fine-tune T-cell activity and preserve peripheral tolerance (**Figure 1**) (15–19). PD-L1 overexpression, observed in many gynecologic malignancies, enables tumor cells to bind PD-1 on activated T-cells and suppress immune cytotoxicity (18–21). Blocking the PD-1/PD-L1 axis restores T-cell function, promotes tumor recognition, and enhances immune-mediated clearance (22). High PD-L1 expression correlates with improved response to checkpoint blockade, establishing PD-L1 as a predictive biomarker for immunotherapy (23).

Ovarian, cervical, and endometrial cancers show frequent but variable PD-L1 expression (17). Ovarian cancers often display PD-L1 on both tumor cells and TAMs, whereas cervical tumors demonstrate high PD-L1 induction through HPV-related inflammation. Endometrial cancers, especially MSI-H or dMMR subtypes, exhibit strong PD-L1 expression associated with high T-cell infiltration. These differences influence responsiveness to PD-1 blockade and guide patient selection.

Therapeutic antibodies targeting the PD-1/PD-L1 axis are primarily derived from IgG subclasses (24). IgG1 antibodies can induce complement-dependent cytotoxicity (CDC) and antibody-dependent cellular cytotoxicity (ADCC), while IgG4 variants minimize these effects (25). The IgG format selection balances immunostimulatory potential with safety (26). Pembrolizumab (IgG4) was approved in 2017 for PD-L1-positive cervical cancer, and dostarlimab (IgG4) in 2021 for dMMR endometrial cancer, emphasizing their therapeutic value in gynecologic oncology.

Recent years have seen a paradigm shift toward immune-based and biologically informed treatments. Pivotal trials—SHAPE, INTERLACE, KEYNOTE-A18, BEATcc, COMPASSION-16, NRG-GY018, RUBY, AtTEnd, DUO-E, LMS-04, PRIMA, DUO-O, ATHENA-combo, and FIRST-ENGOT-OV44—have redefined management across cervical, endometrial, and ovarian cancers (27, 28). Maintenance and combination regimens now incorporate PARP inhibitors, anti-angiogenic agents, and ICIs for improved survival and quality of life.

The approval of niraparib (2019) as first-line maintenance in OC and lenvatinib plus pembrolizumab (2021) for advanced endometrial cancer set new standards of care. The DESTINY-PanTumor02 basket trial further validated trastuzumab deruxtecan for HER2-positive tumors, including gynecologic types. Molecular profiling and biomarker-guided stratification have enhanced precision oncology (27, 28). Trastuzumab deruxtecan, approved in 2022 for HER2-positive solid tumors irrespective of tissue origin, exemplifies antibody-drug conjugates (ADCs) transforming gynecologic oncology (18).

Collectively, these developments mark a transition from conventional chemotherapy to biomarker-driven immunotherapy and ADC approaches. The ongoing priorities are optimizing combination regimens, mitigating resistance, and extending these innovations to diverse histologic subtypes (19, 29). This review therefore examines current therapeutic advances in gynecologic oncology, emphasizing immunotherapy and ADC platforms that improve progression-free survival and reduce systemic toxicity in both early-stage and advanced disease.

## 2 Materials and methods

**Figure 2:**
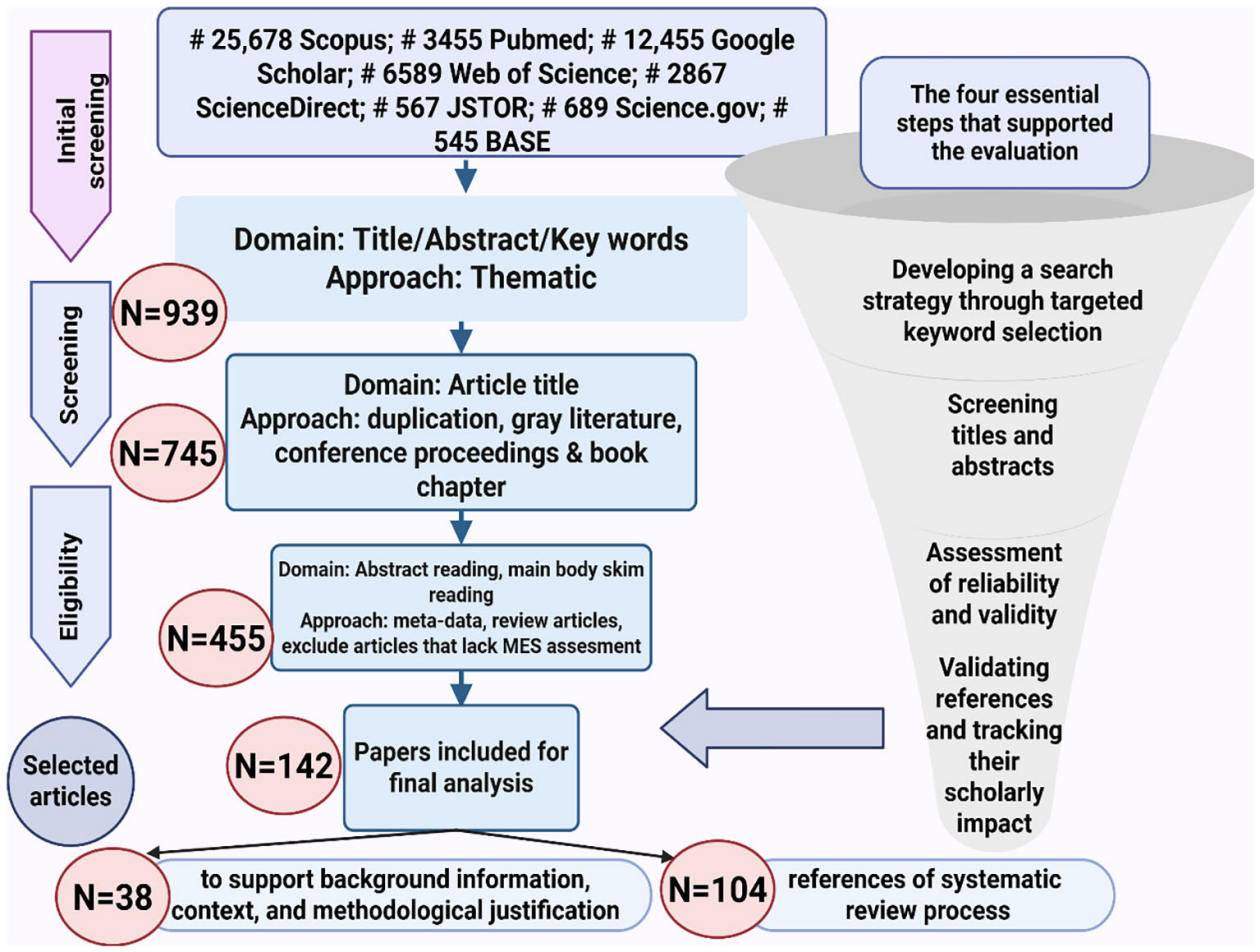
Flowchart of the article selection process and evaluation criteria for the narrative review.

This narrative review followed a structured and transparent literature search to ensure clarity, reproducibility, and comprehensive coverage of recent advances in gynecologic oncology. The main goal was to examine recent progress in systemic therapy, focusing on immunotherapy and ADCs. A structured search strategy was designed that included database selection, keyword definition, and specific inclusion and exclusion criteria (**Figure 2**). The process consisted of four phases: initial search, screening, eligibility assessment, and final inclusion. These stages were applied to identify peer-reviewed studies relevant to immunotherapy, ADCs, and targeted therapies in cervical, endometrial, and ovarian cancers.

A total of 939 records were collected from PubMed, Scopus, Web of Science, ScienceDirect, Google Scholar, JSTOR, Science.gov, and the Bielefeld Academic Search Engine (BASE). After removing duplicates and unrelated records, 745 studies were reviewed by title, and 455 were evaluated by abstract and full text. In the final stage, 142 publications met the eligibility criteria and were included in the qualitative synthesis. Among these, 38 references were used to provide background information and methodological justification, while 104 supported the main analysis of therapeutic evidence and clinical outcomes.

The search was conducted in February 2025 using combinations of the following keywords and Boolean operators: gynecologic oncology, cervical cancer, endometrial cancer, ovarian cancer, immune checkpoint inhibitors, PD-1, PD-L1, antibody–drug conjugates, trastuzumab deruxtecan, clinical trials, targeted therapy, and novel therapies. Only English-language, peer-reviewed publications from 2010 to 2025 were included. Editorials, letters, brief communications, and purely theoretical studies were excluded. Two independent reviewers evaluated each study for relevance and quality, and disagreements were resolved by consensus.

## 3 ADCs: a new frontier in cervical, endometrial, and OC therapy

ADCs have surfaced as a promising therapeutic approach in gynecologic oncology by selectively delivering potent cytotoxic agents to tumor cells while limiting off-target toxicity. In cervical cancer, the ADC tisotumab vedotin (TV) targeting tissue factor (TF) has gained FDA approval based on the innovaTV 204 trial, which showed durable responses in patients with recurrent or metastatic disease after standard chemotherapy failure. In endometrial cancer, agents such as trastuzumab deruxtecan targeting HER2 are under active investigation, especially in tumors with HER2 overexpression (30). Preliminary studies suggest these ADCs may work synergistically with immune checkpoint inhibitors in biomarker-selected subtypes of endometrial carcinoma. In OC, mirvetuximab soravtansine, which targets folate receptor alpha (FRα), has demonstrated encouraging clinical efficacy in platinum-resistant cases. Based on results from the SORAYA trial, this ADC received accelerated FDA approval and is currently under evaluation in combination regimens (31, 32).

Across gynecologic malignancies, current ADCs differ in antigen targets, mechanisms, and payload types. For instance, TF, HER2, and FRα remain the principal targets, while monomethyl auristatin E (MMAE), exatecan, and DM4 are common cytotoxic warheads. Structurally, most agents are IgG1-based conjugates with cleavable linkers. These distinctions, reflected in both approved and investigational compounds, underscore the importance of molecular profiling and tumor-specific antigen expression in optimizing ADC efficacy (**Table 1**).

**Table 1:** Summary of approved and investigational ADCs and ICIs in gynecologic cancers (27, 28).

Tumor type	Target	Agent	Drug type	FDA/EMA approval	Trial phase/notes	Adverse events/Side effect profile
Cervical	TF	Tisotumab vedotin	ADC	FDA and EMA approved for recurrent or metastatic cervical cancer after platinum failure (2L)	innovaTV 204 (Ph II), innovaTV 301 (Ph III)	Ocular toxicity, peripheral neuropathy, epistaxis, nausea
Cervical	PD-1	Pembrolizumab	ICI	FDA and EMA approved for PD-L1 positive persistent, recurrent, or metastatic cervical cancer (1L and 2L)	KEYNOTE-826 (Ph III), KEYNOTE-158 (Ph II)	Fatigue, rash, diarrhea, immune-related AEs
Cervical	PD-L1	Atezolizumab	ICI	No FDA/EMA approval for cervical cancer; investigational	BEATcc (Ph III, combo with bevacizumab)	Infusion reaction, fatigue, immune-related AEs
Cervical	HER2	Trastuzumab deruxtecan	ADC	Not FDA/EMA approved; NCCN Compendium listed for HER2 positive cervical cancer	DESTINY-PanTumor02 (Ph II), CDR 595	Interstitial lung disease, nausea, myelosuppression
Cervical	PD-1 + CTLA-4	Cadonilimab	Bispecific ICI	Not FDA/EMA approved; investigational	ONO-4686 (Ph I/II), dual checkpoint blockade PD-1/CTLA-4	Hepatitis, colitis, rash, thyroiditis
Cervical	Trop-2	Sacituzumab govitecan	ADC	Not FDA/EMA approved; investigational	GOG 3029 (Ph II), ORR 25.9%	Diarrhea, neutropenia, nausea
Cervical	PD-L1	Durvalumab	ICI	Not FDA/EMA approved; investigational	BEATcc (Ph III), PFS benefit	Immune-related AEs (pneumonitis, thyroiditis)
Cervical	CTLA-4	Ipilimumab	ICI	Not FDA/EMA approved; investigational	Combo regimens	Colitis, dermatitis, endocrinopathy
Ovarian	Folate Receptor α	Mirvetuximab soravtansine	ADC	FDA and EMA approved for FRα-high platinum-resistant ovarian cancer (2L+)	SORAYA (Ph III), FRα-targeted	Vision issues, keratopathy, fatigue
Ovarian	NaPi2b	Upifitamab rilsodotin (XMT-1536)	ADC	No FDA/EMA approval; investigational	NaPi2b+DLBCL, Ph I/II	Fatigue, nausea, pneumonitis
Ovarian	PD-L1	Durvalumab	ICI	No FDA/EMA approval; investigational	DUO-O (Ph III), combo with durvalumab + bevacizumab	Fatigue, immune-mediated AEs
Ovarian	PD-1	Nivolumab	ICI	No FDA/EMA approval; investigational	ATHENA Combo (Ph III), no PFS benefit	Immune-mediated pneumonitis, colitis
Ovarian	PD-1	Pembrolizumab	ICI	FDA tumor-agnostic approval for MSI-H/dMMR tumors; no ovarian-specific approval. EMA similar.	KEYNOTE-100, ORR <10% in clear OC	Fatigue, diarrhea, thyroid dysfunction
Ovarian	PD-L1	Atezolizumab	ICI	No FDA/EMA approval for ovarian cancer; investigational	IMagyn050 (Ph III), no benefit	Immune AEs, neutropenia, fatigue
Endometrial	PD-1	Dostarlimab	ICI	FDA and EMA approved for dMMR recurrent or advanced EC after platinum	RUBY Part 1 (Ph III), GARNET (Ph I)	Immune AEs: colitis, rash, hypothyroidism
Endometrial	PD-1	Pembrolizumab	ICI	FDA approved for dMMR/MSI-H EC; FDA approved with lenvatinib for pMMR EC after platinum; EMA similar	KEYNOTE-775 (Ph III)	Fatigue, pruritus, thyroiditis
Endometrial	PD-L1	Atezolizumab	ICI	No FDA/EMA approval; investigational	DUO-E (Ph III), OS benefit in dMMR EC	Immune-related AEs, anemia
Endometrial	Trop-2	Sacituzumab govitecan	ADC	No FDA/EMA approval; investigational	Exploratory, ongoing	Neutropenia, diarrhea, alopecia
Endometrial	HER2	Trastuzumab deruxtecan	ADC	No FDA/EMA approval; investigational	DESTINY-PanTumor02 (Ph II), ORR 57.5%	Myelosuppression, nausea, ILD

## 4 Cervical cancer

### 4.1 Early-stage cervical cancer

Early-stage cervical cancer, classified as FIGO 2009 stages IA1–IB1, comprises tumors confined to the cervix with limited local invasion (33). Screening and HPV vaccination have substantially increased detection at these stages. The standard treatment has traditionally been radical hysterectomy, a procedure associated with considerable morbidity, including urinary, sexual, and bowel dysfunction caused by extensive parametrial dissection (34). Because parametrial invasion occurs in less than 1% of tumors ≤2 cm, less aggressive surgery has been investigated to reduce treatment-related complications without compromising oncologic control (35).

The SHAPE trial was a phase III randomized non-inferiority study comparing simple hysterectomy with radical hysterectomy in patients with low-risk IA2 to IB1 tumors smaller than 2 cm with limited stromal invasion (**Table 2**) (10, 33–35). Among 700 participants, the 3-year pelvic recurrence rates were similar (2.52% for simple hysterectomy versus 2.17% for radical hysterectomy; HR = 1.01; 95% CI 0.42–2.44). Simple hysterectomy was associated with fewer urinary and sexual complications, which supports de-escalation in appropriately selected patients (34, 35, 46, 47).

**Table 2:** Major clinical trials evaluating surgical and adjuvant treatment strategies in early-stage cervical cancer.

Study	Goals and importance	Design	Number of patients	Inclusion criteria	Intervention	Control	Primary endpoint	Key findings
SHAPE (35–37)	To validate if less radical surgery can safely replace radical hysterectomy in small-volume disease	Phase III, randomized, noninferiority	700	Stage IA2 or IB1 (<2 cm), <10 mm invasion or <50% stromal invasion, no lymph node metastasis	Extrafascial simple hysterectomy	Type II radical hysterectomy	3-year pelvic recurrence	Pelvic recurrence similar (2.52% vs 2.17%), HR = 1.12; 95% CI = 0.47–2.67. Fewer urinary complications
RTOG 0724/GOG-0724 (38–40)	To evaluate if adjuvant chemotherapy improves outcomes after chemoradiation in node-positive or margin-positive early-stage cancer	Phase III, randomized, open-label	235	Stage IA2, IB, IIA; Positive nodes or positive parametrial margins after surgery	CRT/VBT + adjuvant TC (paclitaxel/cisplatin) for 4 cycles	CRT/VBT alone	DFS	No significant difference in DFS or OS; 4-year DFS 76.2% vs 76.9%
SENTIX (41, 42)	To validate SLN mapping as a safe alternative to full lymphadenectomy in early-stage cervical cancer	Prospective, observational	594	Stage IA1 (LVSI) – IB1; Squamous cell carcinoma or adenocarcinoma usual type	Bilateral SLN mapping (type B/C radical hysterectomy)	Not available	2-year DFS	2-year DFS 93.3%; OS 97.9%; supports SLN mapping as safe
LACC (43, 44)	To compare MIS vs open surgery in radical hysterectomy; changed global practice guidelines	Phase III, randomized, controlled	631	Early-stage cervical cancer (IA1 with LVSI, IA2, IB1)	Minimally invasive radical hysterectomy	Open radical hysterectomy	DFS & OS	MIS associated with worse DFS (86.0% vs 96.5%) and OS (93.8% vs 99.0%)
ConCerv (45)	To explore microinvasive cervical cancer	Phase II, prospective	100	Stage IA2–IB1 (≤2 cm)	Conservative surgery	None (single-arm)	RFS	Recurrence rate below 5% in well-selected patients

CRT, chemoradiotherapy; VBT, vaginal brachytherapy; PFS, progression-free survival; OS, overall survival; TC, paclitaxel and cisplatin; RT, radiotherapy; PD-1, programmed cell death protein 1; CPS, combined positive score; HR, hazard ratio; IHC, immunohistochemistry; MMAE, monomethyl auristatin E; HER2, human epidermal growth factor receptor 2; irAE, immune-related adverse event; SLN, sentinel lymph node; LVSI, lymphovascular space invasion; MIS, minimally invasive surgery; DFS, disease-free survival; RH, radical hysterectomy.

Additional analyses demonstrated comparable recurrence rates between minimally invasive and open simple hysterectomy (4.3% versus 5.3%), although these were *post-hoc* comparisons (47, 48). Prior evidence from the LACC trial revealed inferior disease-free survival and overall survival for minimally invasive radical hysterectomy compared with open surgery (48–50). These results emphasize that surgical approach selection requires careful consideration. Furthermore, the ConCerv study demonstrated that conservative surgery may be safe for microinvasive tumors measuring 2 cm or smaller, particularly in node-negative and LVSI-negative patients (36, 49, 50). Collectively, SHAPE, LACC, and ConCerv support a trend toward personalized and less invasive surgical strategies in early-stage cervical cancer.

While surgery remains the foundation of treatment in early-stage disease, the therapeutic landscape is shifting toward earlier integration of systemic and immune-based approaches. Immunotherapy and antibody–drug conjugates (ADCs) have shown effectiveness in advanced cervical cancer and are being explored in neoadjuvant and adjuvant settings. Agents under investigation include pembrolizumab and nivolumab (PD-1 inhibitors) and tisotumab vedotin (tissue factor–directed ADC) (32). These agents may enhance tumor response, potentially reducing the need for extensive surgery or adjuvant chemoradiation. Their eventual incorporation into first-line management could help decrease long-term morbidity while maintaining oncologic safety.

Evidence from SHAPE, RTOG 0724, SENTIX, LACC, and ConCerv demonstrates a coordinated shift toward individualized and less aggressive surgical interventions in early-stage cervical cancer without compromising survival (39–43). SHAPE confirmed the safety of simple hysterectomy in low-risk disease, and SENTIX supported sentinel lymph node mapping as an effective strategy that avoids full lymphadenectomy. In contrast, LACC identified limitations of minimally invasive radical hysterectomy, and ConCerv validated conservative, fertility-preserving approaches for select patients. The collective evidence underscores the importance of selecting patients based on tumor size, lymphovascular invasion, and nodal status. Looking forward, integration of minimally invasive techniques with immunotherapy, neoadjuvant immune priming, and patient-reported outcomes may further optimize survival and quality of life in early-stage disease (44, 45).

### 4.2 Locally advanced cervical cancer

Locally advanced cervical cancer, defined as FIGO stages IB2 to IVA, remains a significant therapeutic challenge even though concurrent chemoradiotherapy is the established standard of care (43). Survival rates plateau at approximately 60 percent, which underscores the need for treatment intensification. Recent clinical research has focused on improving survival outcomes by evaluating first-line systemic strategies, including induction chemotherapy, concurrent or maintenance immunotherapy, and adjuvant chemotherapy. Additional goals include refining radiotherapy techniques and identifying patient subgroups most likely to benefit from personalized therapy intensification. Modern trials also emphasize reduction of late toxicity and preservation of quality of life while maintaining durable disease control (**Table 3**).

**Table 3:** Clinical trials in locally advanced cervical cancer.

Study	Goals and importance	Design	Number of patients	Inclusion criteria	Intervention	Control	Primary endpoint	Key findings
INTERLACE (51)	To assess if induction chemotherapy before CRT improves outcomes.	Phase III, randomized, open-label	500	Stage IB1 (node+), IB2, II, IIIB, IVA; squamous, adeno, adenosquamous carcinoma; no aortic node involvement	Weekly TC for 6 weeks then CRT	CRT alone	PFS and OS	Improved 5-year PFS (72 percent vs 64 percent; HR = 0.65; 95 percent CI = 0.46 to 0.91) and OS (80 percent vs 72 percent; HR = 0.60; 95 percent CI = 0.40 to 0.91)
KEYNOTE-A18 (51)	To evaluate pembrolizumab combined with CRT/VBT for high-risk locally advanced disease	Phase III, randomized, double-blind	1,060	Stage IB2–IIB (node+) or III–IVA cervical cancer	Pembrolizumab + CRT/VBT → maintenance pembrolizumab	Placebo + CRT/VBT → maintenance placebo	PFS and OS	Improved 3-year PFS (69.3 percent vs 56.9 percent; HR = 0.68; 95 percent CI = 0.56 to 0.84) and OS (82.6 percent vs 74.8 percent; HR = 0.67; 95 percent CI = 0.50 to 0.90)
CC3 (52)	To evaluate nimotuzumab combined with CRT/VBT	Phase III, randomized, open-label	286	Stage IB3–IVA, squamous type, measurable disease	Nimotuzumab + CRT/VBT	CRT/VBT alone	3-year PFS	No improvement in 3-year PFS or OS
OUTBACK (53)	To determine if adjuvant chemotherapy improves OS after CRT	Phase III, randomized, open-label	919	FIGO 2008 stage IB1 (node+), IB2, II, IIIB, IVA	CRT followed by adjuvant chemotherapy (carboplatin + paclitaxel)	CRT alone	OS	No survival benefit with adjuvant chemotherapy after standard CRT
CALLA (54)	To assess benefit of durvalumab with CRT in locally advanced disease	Phase III, randomized, double-blind	770	Newly diagnosed FIGO 2009 stage IB2–IVA cervical cancer	CRT + durvalumab → maintenance durvalumab	CRT + placebo → maintenance placebo	PFS	Durvalumab added to CRT did not significantly improve PFS
GOG-9929 (55)	To explore sequential CRT and immunotherapy	Phase I/II, non-randomized	34	Stage IB2–IIB, node-positive cervical cancer	CRT followed by ipilimumab	None (single-arm)	Safety and immune response	Ipilimumab was safe and induced immune activation but survival benefit is unknown

CRT, chemoradiotherapy; VBT, vaginal brachytherapy; PFS, progression-free survival; OS, overall survival; TC, paclitaxel and carboplatin; RT, radiotherapy; PD-1, programmed cell death protein 1; CPS, combined positive score; HR, hazard ratio; IHC, immunohistochemistry; FDA, U.S. Food and Drug Administration; ADC, antibody–drug conjugate; MMAE, monomethyl auristatin E; HER2, human epidermal growth factor receptor 2; irAE, immune-related adverse event.

One of the most influential first-line studies is INTERLACE, a phase III open-label randomized trial evaluating the addition of weekly induction chemotherapy before concurrent chemoradiotherapy (51). Patients with FIGO stage IB1 node-positive, IB2, II, IIIB, and IVA disease were treated with weekly paclitaxel and carboplatin for six weeks before standard cisplatin-based chemoradiotherapy. The induction regimen significantly improved five-year progression-free survival (72 percent vs 64 percent; HR = 0.65; 95 percent CI = 0.46 to 0.91) and overall survival (80 percent vs 72 percent; HR = 0.60; 95 percent CI = 0.40 to 0.91; p = 0.015). Although promising, the trial has limitations related to its younger patient population, limited use of IMRT, and exclusion of para-aortic nodal disease, which restrict generalizability (51).

The KEYNOTE-A18 trial introduced a major breakthrough by assessing pembrolizumab added to chemoradiotherapy (52). This phase III double-blind trial enrolled 1,060 patients with FIGO 2014 stage IB2 to IIB node-positive or stage III to IVA cervical cancer. Pembrolizumab was administered concurrently with chemoradiotherapy followed by maintenance therapy. The second interim analysis demonstrated clinically and statistically significant improvements in progression-free survival (69.3 percent vs 56.9 percent; HR = 0.68; 95 percent CI = 0.56 to 0.84; p < 0.001) and overall survival (82.6 percent vs 74.8 percent; HR = 0.67; 95 percent CI = 0.50 to 0.90; p = 0.004). KEYNOTE-A18 is the first phase III study to show a survival advantage with immunotherapy in locally advanced cervical cancer, supported by rapid global enrollment and modern radiotherapy delivery (52).

The OUTBACK trial tested whether adjuvant chemotherapy after chemoradiotherapy improves overall survival (53). This phase III randomized open-label study included patients with FIGO 2008 stage IB1 node-positive to stage IVA disease. Despite a strong biological rationale for cytotoxic intensification, the addition of carboplatin and paclitaxel after chemoradiotherapy did not provide any survival benefit, and overall survival was similar between the treatment groups (53). These results indicate that post-chemoradiotherapy cytotoxic therapy is unlikely to improve outcomes.

The CALLA trial assessed concurrent and maintenance durvalumab with chemoradiotherapy (54). This phase III randomized double-blind study included patients with FIGO 2009 stage IB2 to IVA cervical cancer. Unlike KEYNOTE-A18, CALLA did not show significant improvement in progression-free survival despite a robust immunologic rationale for PD-L1 inhibition during chemoradiation. Several factors may have contributed to the negative outcome, including a high percentage of stage IIIB disease, heterogeneity in PD-L1 expression, and a relatively short median follow-up of 18.5 months (54).

Further insight comes from GOG-9929, a phase I/II non-randomized study evaluating sequential ipilimumab after chemoradiotherapy in stage IB2 to IIB node-positive cervical cancer. Up to four cycles of ipilimumab were administered, showing acceptable safety and evidence of T cell activation (55). Although not powered to assess survival outcomes, GOG-9929 supports biologic feasibility of post-CRT checkpoint blockade and informs ongoing dual checkpoint studies.

A complementary study, the CALLA trial, also assessed combining immunotherapy with CRT, but with different results (54). This Phase III randomized double-blind trial involved patients with newly diagnosed FIGO 2009 stage IB2–IVA cervical cancer who received CRT plus either durvalumab (PD-L1 inhibitor) or placebo. No significant improvement in PFS was observed, despite a strong biological rationale for PD-L1 inhibition during CRT (54). Possible contributing factors included a high proportion of stage IIIB cases, ethnic diversity, variable PD-L1 expression, and a short median follow-up (18.5 months), potentially limiting detection of long-term immunotherapy benefits (54).

To better understand immunotherapy sequencing, the GOG-9929 trial investigated ipilimumab, a CTLA-4 inhibitor, given after CRT. This Phase I/II non-randomized study enrolled patients with FIGO stage IB2–IIB node-positive cervical cancer. Patients received up to four doses of ipilimumab following CRT. The trial showed that sequential ipilimumab was safe and biologically active, with increased T-cell activation markers (55). While not designed to measure survival outcomes, the trial provided key immunologic insights and laid the groundwork for ongoing studies on dual checkpoint blockade with CRT. Recent trials—including INTERLACE, KEYNOTE-A18, OUTBACK, CALLA, and GOG-9929—highlight evolving strategies for treating locally advanced cervical cancer, but also point to persistent challenges. Among the most promising results, INTERLACE demonstrated that induction chemotherapy before CRT significantly improved survival. A brief weekly paclitaxel-carboplatin regimen resulted in 5-year PFS of 72% versus 64% in the CRT-only group, and 5-year OS of 80% vs. 72% (53). These outcomes support the rationale that tumor debulking enhances radiosensitivity and controls micrometastatic disease. The protocol’s short duration and high compliance further strengthen its clinical utility.

However, limitations of INTERLACE must be acknowledged. Participants were generally younger and healthier than typical real-world LACC patients (53). In addition, many received 3D-CRT instead of IMRT, possibly underestimating the potential of modern radiotherapy (53). The exclusion of patients with para-aortic nodal involvement also narrows its applicability. Building on intensification strategies, KEYNOTE-A18 marked a breakthrough by adding pembrolizumab to CRT. It was the first Phase III trial to show a statistically significant OS benefit from integrating a PD-1 inhibitor into standard treatment for locally advanced cervical cancer. Patients receiving concurrent and maintenance pembrolizumab achieved a 3-year PFS of 69.3%, compared to 56.9% in the control group. Other strengths included the use of advanced radiotherapy techniques and rapid global recruitment, supporting the generalizability of findings. However, several limitations must be considered. Despite the observed survival benefit, concerns remain about the cost and accessibility of pembrolizumab in low- and middle-income countries, where cervical cancer is most prevalent. Additionally, subgroup analyses suggested greater benefit in non-White populations, though these findings should be interpreted with caution due to small sample sizes (54). In contrast to these promising results, the OUTBACK trial illustrates the challenges of adjuvant chemotherapy after CRT. Although the rationale for systemic intensification to eliminate micrometastases is strong, OUTBACK demonstrated no survival improvement with the addition of carboplatin-paclitaxel after CRT (55). A major strength of the trial was its robust design and comprehensive follow-up, which confirmed that adjuvant cytotoxic chemotherapy offers no additional survival benefit after CRT.

A supporting study, CALLA, also emphasized the complexities of combining immunotherapy with CRT (54). Unlike KEYNOTE-A18, the CALLA trial, which evaluated durvalumab (a PD-L1 inhibitor) plus CRT, showed no statistically significant improvement in PFS. Nonetheless, CALLA’s strengths included rigorous methodology and broad international representation, providing insights into real-world applicability (56). It also highlighted potential limitations of PD-L1 blockade during CRT. Several factors may explain CALLA’s lack of efficacy. First, the study included a higher proportion of FIGO stage IIIB patients, suggesting a greater tumor burden (54). Second, PD-L1 expression was not used as a stratification factor, possibly including many patients less likely to respond. Lastly, the short median follow-up (18.5 months) may have been insufficient to capture delayed immunologic effects (51–55).

Further investigation into sequencing strategies was explored in the GOG-9929 trial, which assessed ipilimumab (CTLA-4 inhibitor) after CRT. A key finding was the biological feasibility of this approach, shown by increased T-cell activation. Importantly, the safety profile was acceptable, with no unexpected immune-related toxicities. However, the non-randomized design and small sample size limit conclusions about clinical benefit. Without a comparator arm, it remains unclear whether immune activation translates to improved survival. Still, GOG-9929 laid the groundwork for current studies on dual checkpoint inhibition and optimal immunotherapy timing (27–29). Taken together, these trials suggest key insights. Concurrent or maintenance immunotherapy appears more promising than adjuvant chemotherapy in LACC. The contrasting results of KEYNOTE-A18 (positive) and OUTBACK (negative) indicate that immunologic synergy during CRT is likely more effective than sequential cytotoxic therapy. Future trials should prioritize biomarker discovery—including PD-L1 expression, tumor mutational burden, and circulating tumor DNA—to enable more personalized treatments. Additionally, the cost-effectiveness and accessibility of therapies like pembrolizumab must be considered, especially in resource-limited settings (27, 28).

Crucially, trial design should reflect real-world complexities, including differences in tumor burden, radiotherapy access, and ethnic diversity. While KEYNOTE-A18 set a benchmark for global immunotherapy research, the negative outcomes of CALLA and OUTBACK have provided important lessons that will shape future directions (51). In summary, recent trials in locally advanced cervical cancer have revealed both substantial progress and ongoing challenges. Lessons learned from INTERLACE, KEYNOTE-A18, OUTBACK, CALLA, and GOG-9929 now guide future research aimed at improving outcomes for women worldwide.

### 4.3 Immunotherapy-based combination strategies in metastatic, persistent, and recurrent cervical cancer

Metastatic and recurrent cervical cancer continues to pose a significant clinical challenge, with historically limited treatment options and poor long-term survival. Over the last decade, the introduction of targeted agents, antibody drug conjugates (ADCs), and immune checkpoint inhibitors has markedly reshaped the therapeutic landscape. The central aims of contemporary clinical trials in this setting are to prolong progression-free survival (PFS) and overall survival (OS), optimize first-line systemic treatment, and provide effective later-line options for patients who progress after platinum-based chemotherapy. Key landmark studies, including GOG-240, KEYNOTE-826, BEATcc, COMPASSION-16, EMPOWER-Cervical 1, and SKB264-II-06, have collectively refined systemic therapy strategies for this high-risk population (**Table 4**) (51–55).

**Table 4:** Clinical trials in locally advanced cervical cancer.

Study	Goals and importance	Design	Number of patients	Inclusion criteria	Intervention	Control	Primary endpoint	Key findings
BEATcc (51)	To assess whether atezolizumab + bevacizumab + chemotherapy improves outcomes	Phase III, randomized, open-label	410	Metastatic, persistent, or recurrent cervical cancer, no prior systemic therapy, measurable disease	Atezolizumab + TC or TP + bevacizumab, maintenance atezolizumab and bevacizumab	TC or TP + bevacizumab, maintenance bevacizumab	PFS and OS	Improved median PFS (13.7 vs 10.4 months; HR = 0.62; 95 percent CI = 0.49 to 0.78) and median OS (32.1 vs 22.8 months; HR = 0.68; 95 percent CI = 0.52 to 0.88)
SKB264-II-06 (52)	To evaluate Sacituzumab-TMT + pembrolizumab in platinum-refractory disease	Phase II, open-label, basket trial	40	Recurrent/metastatic cervical cancer, progressed after platinum-doublet therapy	Sacituzumab-TMT + pembrolizumab every 6 weeks	None (single-arm)	Safety, ORR	6-month PFS rate 65.7 percent; ORR 57.9 percent
COMPASSION-16 (53)	To evaluate cadonilimab + chemotherapy ± bevacizumab	Phase III, randomized, double-blind	445	Metastatic, persistent, or recurrent cervical cancer, no prior systemic therapy	Cadonilimab + TC/TP ± bevacizumab	Placebo + TC/TP ± bevacizumab	PFS and OS	Improved median PFS (12.7 vs 8.1 months; HR = 0.62; 95 percent CI = 0.49 to 0.80) with OS benefit observed
GOG-240 (54)	First trial to show survival benefit with antiangiogenic therapy	Phase III, randomized, open-label	452	Recurrent/persistent/metastatic cervical cancer	TC/TP + bevacizumab	TC/TP alone	OS	Bevacizumab addition improved OS (17.0 vs 13.3 months; HR = 0.71; p = 0.0035)
KEYNOTE-826 (55)	To assess pembrolizumab + chemotherapy ± bevacizumab	Phase III, randomized, double-blind	617	Persistent, recurrent, or metastatic cervical cancer	Pembrolizumab + TC/TP ± bevacizumab	Placebo + TC/TP ± bevacizumab	PFS and OS	Pembrolizumab addition improved PFS (10.4 vs 8.2 months) and 24-month OS (53 percent vs 41.7 percent)
EMPOWER-Cervical 1 (53)	First PD-1 monotherapy to show survival benefit in 2nd-line setting	Phase III, randomized, open-label	608	Recurrent/metastatic cervical cancer after platinum-based chemotherapy	Cemiplimab	Investigator’s choice chemotherapy	OS	Cemiplimab improved OS compared with chemotherapy (12.0 vs 8.5 months; HR = 0.69; 95 percent CI = 0.56 to 0.84)

AUC, area under the curve; BICR, blinded independent central review; CRT, chemoradiotherapy; HER2, human epidermal growth factor receptor 2; IMRT, intensity-modulated radiotherapy; NR, not reached; ORR, objective response rate; OS, overall survival; PFS, progression-free survival; PD-1, programmed cell death protein 1; PD-L1, programmed death-ligand 1; Sac-TMT, sacituzumab govitecan conjugated with a topoisomerase I inhibitor; TC, paclitaxel and carboplatin; TP, paclitaxel and cisplatin; VBT, vaginal brachytherapy.

In the first-line setting, the GOG-240 trial was pivotal, as it first established the value of adding antiangiogenic therapy to chemotherapy in recurrent and metastatic disease (54). This phase III randomized open-label study demonstrated that the addition of bevacizumab to a paclitaxel plus cisplatin or carboplatin backbone improved OS from 13.3 to 17.0 months (HR = 0.71; p = 0.0035), thereby defining a new standard of care and providing the rationale for subsequent chemoimmunotherapy and chemo–anti-VEGF combinations. Building on this foundation, KEYNOTE-826 evaluated pembrolizumab, a PD-1 inhibitor, in combination with platinum-based chemotherapy with or without bevacizumab in persistent, recurrent, or metastatic cervical cancer (55). In this phase III randomized double-blind trial of 617 patients, pembrolizumab-containing regimens significantly improved PFS (10.4 vs 8.2 months) and 24-month OS (53 percent vs 41.7 percent), firmly establishing pembrolizumab plus chemotherapy with or without bevacizumab as a new standard first-line approach for PD-L1-positive disease.

The BEATcc trial further expanded first-line options by assessing the integration of immunotherapy into the bevacizumab-containing platform (51). This phase III randomized open-label study evaluated atezolizumab plus paclitaxel and cisplatin or carboplatin with bevacizumab versus chemotherapy plus bevacizumab alone in metastatic, persistent, or recurrent cervical cancer. BEATcc showed a clinically meaningful improvement in median PFS (13.7 vs 10.4 months; HR = 0.62; 95 percent CI = 0.49 to 0.78; p < 0.0001) and median OS (32.1 vs 22.8 months; HR = 0.68; 95 percent CI = 0.52 to 0.88; p = 0.0046) (51). Importantly, PD-L1 status was not used as an eligibility criterion, which broadens the applicability of its findings to a wider patient population.

Subsequently, the COMPASSION-16 study reinforced the benefit of immunotherapy-based combinations as first-line therapy (53). This phase III randomized double-blind trial evaluated cadonilimab, a bispecific antibody targeting PD-1 and CTLA-4, in combination with chemotherapy with or without bevacizumab in patients with metastatic, persistent, or recurrent cervical cancer. Cadonilimab significantly improved median PFS (12.7 vs 8.1 months; HR = 0.62; 95 percent CI = 0.49 to 0.80; p < 0.0001) and showed clinical benefit even in PD-L1-negative patients, likely due to its dual checkpoint inhibition mechanism (53). The inclusion of a large Asian population enhances the relevance of COMPASSION-16 across diverse global settings.

Later-line immunotherapy strategies have also reshaped management. The EMPOWER-Cervical 1 trial evaluated cemiplimab monotherapy in patients with recurrent or metastatic cervical cancer after platinum-based chemotherapy failure (53). This phase III randomized open-label study demonstrated a significant OS benefit for cemiplimab over investigator’s choice chemotherapy (12.0 vs 8.5 months; HR = 0.69; 95 percent CI = 0.56 to 0.84; p < 0.001), representing the first PD-1 monotherapy to show a survival advantage in the second-line setting and providing an important option for patients who have exhausted standard chemotherapy.

ADCs in combination with immunotherapy represent an emerging strategy, particularly for later-line treatment. The SKB264-II-06 trial, a phase II open-label basket study, investigated sacituzumab-TMT (an ADC based on sacituzumab govitecan conjugated with a topoisomerase I inhibitor) combined with pembrolizumab in patients with recurrent or metastatic cervical cancer who had progressed after one or two prior systemic regimens (52). Among this platinum-refractory cohort, the regimen achieved a 6-month PFS rate of 65.7 percent and an objective response rate (ORR) of 57.9 percent (52). Although limited by its single-arm design and relatively small sample size of 40 patients, SKB264-II-06 suggests that ADC plus checkpoint inhibitor combinations may provide meaningful activity in heavily pretreated disease and warrants confirmation in larger randomized trials.

Recent clinical trials such as BEATcc (51), KEYNOTE-826 (55), and COMPASSION-16 (53) have Taken together, these trials define a coherent framework in which first-line therapy for metastatic, persistent, and recurrent cervical cancer increasingly relies on chemoimmunotherapy and antiangiogenic combinations, while later-line strategies incorporate PD-1 monotherapy and emerging ADC plus immunotherapy regimens. BEATcc and KEYNOTE-826 demonstrate that adding checkpoint inhibition to platinum-based chemotherapy, often on a bevacizumab backbone, can significantly improve PFS and OS. COMPASSION-16 further suggests that bispecific blockade of PD-1 and CTLA-4 may extend benefit to patients with PD-L1-negative disease. EMPOWER-Cervical 1 shows that cemiplimab monotherapy offers a survival advantage after platinum failure, and SKB264-II-06 highlights the potential for ADC plus PD-1 combinations in heavily pretreated patients (51–55).

Despite these advances, several limitations must be acknowledged, including selection of predominantly PD-L1-positive or treatment-naïve populations, modest sample sizes in early-phase ADC studies, and high costs that restrict access in low- and middle-income countries. Future research should prioritize broader real-world representation, formal cost-effectiveness analyses, and biomarker-driven stratification using PD-L1 expression, tumor mutational burden, and circulating tumor DNA to refine patient selection (54–59). Longer follow-up is also required to clarify the durability of responses and late toxicity profiles associated with prolonged immunotherapy and ADC exposure. As ongoing and upcoming trials build on the lessons from GOG-240, KEYNOTE-826, BEATcc, COMPASSION-16, EMPOWER-Cervical 1, and SKB264-II-06, a more personalized and globally accessible treatment paradigm for metastatic and recurrent cervical cancer may be achievable, with the ultimate goal of improving survival and quality of life for women worldwide (60, 61).

## 5 ADC advances in cervical cancer

ADCs have expanded options for recurrent or metastatic cervical cancer, where historical second-line chemotherapy produced modest response rates of 0–6% (62–66). TV an antibody–drug conjugate targeting tissue factor, represents the most advanced ADC in this setting. TV received regulatory approval based on the phase II innovaTV 204 trial, which evaluated 102 previously treated patients and demonstrated an objective response rate (ORR) of 24% and a median overall survival (OS) of 12.1 months (54, 65, 67). Ocular adverse events were characteristic but manageable with prophylaxis and close monitoring (68, 69).

The phase III innovaTV 301 trial subsequently confirmed these findings, randomizing 502 patients with recurrent or metastatic cervical cancer to receive TV versus investigator’s choice chemotherapy. TV significantly reduced the risk of death by 30% (HR = 0.70; 95% CI = 0.54–0.89) and improved progression-free survival (HR = 0.67; 95% CI = 0.54–0.82), establishing TV as a preferred second-line therapy for this population (55, 70–72).

Trastuzumab deruxtecan is an ADC for HER2-positive cervical cancer. In DESTINY-PanTumor02, the cervical cancer cohort (n=40) showed an ORR of 50% in a heavily pretreated population, supporting activity in this subgroup (68, 73). Based on these results, trastuzumab deruxtecan appears in the NCCN Compendium for recurrent HER2-positive disease, typically defined by IHC 2+ or 3+ (67).

Together, TV and trastuzumab deruxtecan broaden the systemic therapy landscape in cervical cancer by providing effective options for unselected second-line populations (TV) and HER2-positive subsets (trastuzumab deruxtecan), with benefits demonstrated in prospective trials (67–70, 73).

## 6 Endometrial cancer

Endometrial cancer remains one of the most common gynecologic malignancies worldwide. While early-stage cases are often curable with surgery and adjuvant radiotherapy or chemotherapy, advanced or recurrent disease continues to pose a major clinical challenge. Historically, platinum-based chemotherapy has been the standard of care; however, durable responses are limited, particularly in patients with pMMR tumors. Over the past decade, the emergence of immune checkpoint inhibitors (ICIs), targeted agents and ADCs has transformed the therapeutic landscape by exploiting specific molecular vulnerabilities, offering new options for patients with previously few alternatives (27–29). Key phase III and pivotal phase II studies, including RUBY Part 1, NRG-GY018, AtTEnd, DUO-E, LEAP-001, ENGOT-en9/DUO-O and TOTEM, have evaluated novel combinations in first-line and recurrent settings, aiming to improve PFS, OS and sustained disease control across distinct molecular subtypes (**Table 5**) (74–84).

**Table 5:** Clinical trials evaluating immune checkpoint inhibitors and targeted therapies in advanced or recurrent endometrial cancer (74–80).

Study	Goals and importance	Design	Number of patients	Inclusion criteria	Intervention	Control	Primary endpoint	Key findings
RUBY Part 1 (74)	To evaluate dostarlimab plus chemotherapy in first-line advanced/recurrent endometrial cancer	Phase III, randomized, double-blind	494	Advanced or recurrent EC; prior chemotherapy allowed after ≥6 months	Dostarlimab + carboplatin-paclitaxel	Placebo + carboplatin-paclitaxel	PFS and OS	Dostarlimab plus chemotherapy improved PFS (11.8 vs 7.9 months; HR = 0.64) with an emerging OS benefit in interim analysis
NRG-GY018 (75)	To assess pembrolizumab + chemotherapy across biomarker subgroups	Phase III, randomized, double-blind	816	Advanced or recurrent EC; chemotherapy-naïve or prior therapy ≥12 months before	Pembrolizumab + carboplatin-paclitaxel	Placebo + carboplatin-paclitaxel	PFS in dMMR and pMMR cohorts	Pembrolizumab plus chemotherapy significantly improved PFS in dMMR (HR = 0.30) and pMMR (HR = 0.54) cohorts
AtTEnd (76)	Long-term follow-up of AtTEnd trial patients; To assess pembrolizumab + chemotherapy across biomarker subgroups	Phase III, randomized, double-blind	551	Advanced or recurrent EC; chemotherapy-naïve or prior therapy ≥6 months before	Atezolizumab + carboplatin-paclitaxel	Placebo + carboplatin-paclitaxel	PFS and OS	Atezolizumab plus chemotherapy improved PFS (10.1 vs 8.9 months; HR = 0.74); OS data are maturing with continued follow-up
DUO-E (77)	To evaluate durvalumab ± olaparib with chemotherapy	Phase III, randomized, double-blind	718	Advanced or recurrent EC; chemotherapy-naïve; allowed prior platinum if >12 months	Durvalumab + carboplatin-paclitaxel ± olaparib maintenance	Placebo + carboplatin-paclitaxel ± placebo maintenance	PFS	Durvalumab plus olaparib maintenance improved PFS (15.1 vs 10.2 months; HR = 0.55) and showed an encouraging OS trend
LEAP-001 (78)	To assess pembrolizumab + lenvatinib as first-line therapy	Phase III, randomized, open-label	827	Advanced or recurrent EC; chemotherapy-naïve	Lenvatinib + pembrolizumab	Carboplatin + paclitaxel	PFS and OS	Lenvatinib plus pembrolizumab did not demonstrate noninferiority to chemotherapy in the primary analysis; final results under review
ENGOT-en9/DUO-O (79)	To investigate adding olaparib to durvalumab + chemotherapy maintenance	Phase III, randomized, double-blind	1200	Advanced EC with biomarker selection (HRD, BRCA, dMMR)	Olaparib + durvalumab + carboplatin-paclitaxel	Placebo + durvalumab + carboplatin-paclitaxel	PFS	Olaparib plus durvalumab with chemotherapy improved PFS compared with the durvalumab plus chemotherapy control (HR = 0.42)
TOTEM (80)	Early-phase investigation of ADC in EC	Phase II, single-arm	100	Advanced or recurrent EC; prior therapy	Sacituzumab govitecan	None (single-arm)	ORR	Trop-2 targeted ADC sacituzumab govitecan showed early antitumor activity with clinically meaningful ORR in heavily pretreated patients

ADC, antibody–drug conjugate; AE, adverse event; CARB, carboplatin; CI, confidence interval; CRT, chemoradiotherapy; dMMR, mismatch repair-deficient; EC, endometrial cancer; HR, hazard ratio; ITT, intention-to-treat; IHC, immunohistochemistry; mAb, monoclonal antibody; mOS, median overall survival; mPFS, median progression-free survival; NR, not reached; ORR, objective response rate; OS, overall survival; PFS, progression-free survival; pMMR, mismatch repair-proficient; TF, tissue factor.

The RUBY Part 1 trial marked a major advance by evaluating dostarlimab, a PD-1 inhibitor, with carboplatin–paclitaxel in advanced or recurrent endometrial cancer (74). This randomized phase III study demonstrated significantly improved median PFS (11.8 vs. 7.9 months; HR = 0.64; p < 0.001) and a favorable OS trend (44.6 vs. 28.2 months; HR = 0.69; p = 0.002). The greatest benefit was observed in the mismatch repair-deficient (dMMR) subgroup (HR = 0.28; p < 0.001), underscoring the central role of biomarker-based stratification. Inclusion of patients with carcinosarcomas broadened its applicability to aggressive histologies. Similarly, NRG-GY018 evaluated pembrolizumab plus carboplatin–paclitaxel with prespecified cohorts by dMMR and pMMR status, reporting significant PFS gains in both groups (dMMR HR = 0.30; pMMR HR = 0.54; both p < 0.001), thereby reinforcing checkpoint blockade as a backbone in both biologically favorable and less immunogenic tumors (75). The AtTEnd trial, which assessed the addition of atezolizumab, a PD-L1 inhibitor, to carboplatin–paclitaxel, showed a more modest but clinically relevant PFS benefit (10.1 vs. 8.9 months; HR = 0.74; p = 0.0022), with extended follow-up confirming the advantage and OS data still maturing (76).

Beyond pure chemo–immunotherapy, several studies have integrated ICIs with agents targeting the DNA damage response. The DUO-E trial evaluated durvalumab in combination with carboplatin–paclitaxel followed by maintenance durvalumab with or without the PARP inhibitor olaparib (77). This immune–DNA repair co-targeting approach led to a significant PFS improvement (15.1 vs. 10.2 months; HR = 0.55; p < 0.0001) and an encouraging OS trend (HR = 0.71; p = 0.003), highlighting synergy between checkpoint inhibition and PARP-mediated DNA-damage modulation rather than PARP inhibitor monotherapy (77). In parallel, ENGOT-en9/DUO-O further explored the addition of olaparib to durvalumab plus chemotherapy in biomarker-selected populations with homologous recombination deficiency (HRD) and/or BRCA mutations, demonstrating a robust PFS extension (HR = 0.42; p < 0.0001) compared to chemo-based regimens without olaparib (79). These data support a dual-modality paradigm in HRD-enriched cohorts.

The LEAP-001 trial investigated a chemotherapy-free front-line strategy by comparing pembrolizumab plus lenvatinib, a multi-tyrosine kinase inhibitor, with carboplatin–paclitaxel in advanced or recurrent endometrial cancer (78). Although final noninferiority conclusions are pending and noninferiority was not proven in the primary analysis, this study reflects the growing interest in replacing or delaying cytotoxic chemotherapy with ICI–TKI combinations, particularly in biomarker-positive tumors (78). In the recurrent setting, the KEYNOTE-775 trial provided definitive evidence that pembrolizumab plus lenvatinib significantly improves outcomes in previously treated pMMR advanced endometrial cancer, enhancing median PFS (6.6 vs. 3.8 months; HR = 0.60; p < 0.001) and OS (17.4 vs. 12.0 months; HR = 0.68; p < 0.001) compared with physician’s-choice chemotherapy (83). Given the limited activity of pembrolizumab monotherapy in pMMR tumors, this combination filled a critical treatment gap and led to FDA approval in 2021 for pMMR advanced endometrial cancer following platinum failure (83).

Biomarkers have emerged as key determinants of immunotherapy benefit. The KEYNOTE-158 basket trial included a cohort of patients with endometrial cancer and a broader MSI-H/dMMR population, showing that pembrolizumab monotherapy achieved an objective response rate (ORR) of 48% in MSI-H/dMMR endometrial cancer, with a median PFS of 13.1 months and a median OS not reached (81). These durable responses validated MSI-H/dMMR status as a powerful predictive biomarker and supported tissue-agnostic approvals of PD-1 blockade. Subsequent analyses have confirmed that ICIs achieve response rates of approximately 27–58% in MMR-d/MSI-H endometrial cancer, while integration with chemotherapy or tyrosine kinase inhibitors (TKIs) is redefining standards of care across molecular subsets (82). In NRG-GY018, the magnitude of benefit in both dMMR and pMMR cohorts, together with KEYNOTE-158 data, led to the inclusion of pembrolizumab–chemotherapy regimens as Category I recommendations in the National Comprehensive Cancer Network (NCCN) guidelines for stage III–IV disease (75, 81, 82).

Dostarlimab has also become a central PD-1 inhibitor in this space. In the phase I GARNET trial, dostarlimab-gxly produced an ORR of 42.3% in patients with dMMR recurrent endometrial cancer, with durable responses and a manageable safety profile, prompting accelerated FDA approval for dMMR recurrent disease that had progressed after platinum-based therapy (84). RUBY Part 1 then extended these findings into the frontline setting, where adding dostarlimab to carboplatin–paclitaxel significantly improved PFS in both the overall and dMMR/MSI-H populations, with a 24-month PFS of 61.4% versus 15.7% in the dMMR/MSI-H subgroup (HR = 0.28; p < 0.001) (74, 85). These results established dostarlimab–chemotherapy as a new standard for dMMR advanced or recurrent endometrial cancer and reinforced the need for universal MMR/MSI testing at diagnosis (74, 84, 85).

Durvalumab and atezolizumab provide additional PD-L1-targeted options. As noted above, durvalumab-containing regimens in DUO-E and ENGOT-en9/DUO-O significantly improved PFS in biomarker-selected populations, especially those with HRD or BRCA alterations (77, 79, 86). AtTEnd evaluated atezolizumab plus carboplatin–paclitaxel and demonstrated an overall PFS benefit (HR = 0.74; p = 0.0219), with an even more pronounced advantage in dMMR tumors (HR = 0.36; p = 0.0219), suggesting that multiple PD-1/PD-L1 inhibitors can exploit similar biomarker axes in endometrial cancer (76, 87). Final OS readouts are awaited, but the totality of evidence supports PD-L1 blockade as another pillar of immunotherapy in this disease.

ADCs are rapidly expanding the therapeutic armamentarium. The phase II TOTEM study evaluated sacituzumab govitecan, a Trop-2-directed ADC, in heavily pretreated advanced or recurrent endometrial cancer and demonstrated early signals of activity, with ORR exceeding expectations in this difficult-to-treat population (80). In parallel, trastuzumab deruxtecan, an ADC targeting HER2, showed encouraging results in the DESTINY-PanTumor02 trial, where the endometrial cancer cohort achieved an ORR of 57.5%, substantially higher than historical chemotherapy controls (88). The STATICE trial further extended the utility of trastuzumab deruxtecan to HER2-high and HER2-low uterine carcinosarcomas, reporting ORRs of 54.5% and 70%, respectively, indicating that even low HER2 expression can be therapeutically exploitable, likely due to the bystander killing effect of the payload (89). Collectively, these data support HER2 testing in selected histologies and position ADCs as promising options for recurrent disease.

Given these advances, the management of endometrial cancer is increasingly structured around biomarker-informed algorithms. Routine dMMR/MSI-H testing identifies candidates for PD-1 monotherapy or chemo–immunotherapy, while HER2 assessment guides the use of trastuzumab-based regimens and ADCs in serous and carcinosarcoma histologies. Additional biomarkers, including PD-L1 expression, tumor mutational burden and HRD status, are being refined to personalize the choice between ICI monotherapy, ICI–TKI combinations, and ICI–PARP strategies (74–83, 86–89). Future directions include combining checkpoint inhibitors with anti-angiogenic agents such as lenvatinib, DNA-damage repair modulators such as olaparib, or additional immune checkpoints such as TIGIT and LAG-3, as well as incorporating next-generation sequencing, circulating tumor DNA monitoring and dynamic biomarker tracking to optimize patient selection (77, 79, 83, 86). Overall, immunotherapy and targeted agents have significantly broadened the options for advanced and recurrent endometrial cancer, and ongoing trials such as DUO-E, AtTEnd and DESTINY-PanTumor02 are expected to further advance precision oncology, with the goal of achieving durable disease control and improved survival for all patients (76, 77, 88).

## 7 Ovarian cancer

OC continues to be one of the most lethal gynecologic cancers, as most instances are identified at advanced stages. Even with improvements in surgical methods and chemotherapy based on platinum, the rates of recurrence continue to be elevated, and lasting remission is uncommon. In the last ten years, substantial work has been done to enhance results by incorporating targeted treatments, immune checkpoint blockers, and innovative maintenance approaches. A variety of clinical trials, including DUO-O, ATHENA Combo, PRIMA, NeoPembrOV, ANITA, ATALANTE, NRG-GY005, AGO-OVAR 2.29/ENGOT-ov34, LARA, BrUOG 354, and CARACO, have investigated innovative treatment alternatives (**Table 6**). These studies aim to enhance not only PFS and OS but also to improve patient stratification using molecular biomarkers like HRD and immune profiles. Together, these trials signify a move toward personalized treatment methods intended to enhance results for patients with locally advanced or recurrent OC, providing optimism for more efficient, lasting therapies beyond conventional cytotoxic methods (101).

**Table 6:** Major clinical trials in advanced OC.

Study	Goals and importance	Design	Number of patients	Inclusion criteria	Intervention	Control	Primary endpoint	Key findings
DUO-O (90)	Evaluate durvalumab + olaparib maintenance after chemo-bevacizumab	Phase III, randomized, double-blind	1,130	Stage III–IV high-grade epithelial, non-tBRCAm, no prior systemic therapy	TC + bevacizumab + durvalumab → bevacizumab + durvalumab + olaparib maintenance	TC + bevacizumab + placebo	PFS	Median PFS: 25.1 vs. 19.3 mo; HR = 0.61 (95% CI = 0.51–0.73)
ATHENA (91) Combo (92)	Assess nivolumab + rucaparib maintenance	Phase III, randomized, double-blind	863	Stage III–IV high-grade epithelial, response after first-line platinum	Rucaparib + nivolumab maintenance	Rucaparib + placebo maintenance	PFS	Median PFS: 15.0 vs. 20.2 mo; HR = 1.29 (95% CI = 1.08–1.53)
NeoPembrOV (93)	Neoadjuvant chemoimmunotherapy	Phase II, randomized, open-label	91	Stage IIIC/IV high-grade serous/endometrioid, PCI <30, unresectable upfront	TC + pembrolizumab, maintenance pembrolizumab	TC alone	CRR at IDS, PFS	Median PFS: 19.4 vs. 20.8 mo
ANITA (94)	Evaluate atezolizumab + chemotherapy + niraparib maintenance	Phase III, randomized, double-blind	417	Platinum-sensitive recurrence; TFI ≥6 months	Carboplatin doublet + atezolizumab + niraparib maintenance	Carboplatin doublet + placebo + niraparib maintenance	PFS	Median PFS: 11.2 vs. 10.1 mo
ATALANTE (95)	Evaluate bevacizumab + atezolizumab in recurrence	Phase III, randomized, double-blind	614	Recurrent epithelial non-mucinous OC; 1–2 prior lines	Carboplatin chemo + bevacizumab + atezolizumab maintenance	Carboplatin chemo + bevacizumab	PFS	Median PFS: 13.6 vs. 11.3 mo; HR = 0.83 (p=0.035)
NRG-GY005 (96)	Cediranib + olaparib vs chemo in platinum-resistant OC	Phase II/III, randomized, open-label, superiority	562	Platinum-refractory/resistant high-grade serous/endometrioid	Cediranib + olaparib (or cediranib alone)	Weekly paclitaxel, PLD, or topotecan	PFS, OS	Median PFS: 5.2 vs. 4.3 mo; HR = 0.75
AGO-OVAR 2.29/ENGOT-ov34 (97)	Evaluate atezolizumab + bevacizumab with chemotherapy	Phase III, randomized, double-blind	574	Recurrent platinum-sensitive high-grade OC	PLD/paclitaxel + bevacizumab + atezolizumab	PLD/paclitaxel + bevacizumab	PFS	Median PFS: 6.3 vs. 6.6 mo; HR = 0.88
LARA (98)	Evaluate pembrolizumab + lenvatinib in clear cell OC	Phase II, open-label, two-stage	27	Recurrent clear cell OC of ovary or endometrium	Pembrolizumab + lenvatinib	None	ORR, PFS	ORR at 24 wk: 60%
BrUOG 354 (99)	Nivolumab ± ipilimumab in clear cell OC	Phase II, randomized, two-arm	44	Extra-renal clear cell OC relapse	Nivolumab alone vs Nivolumab + ipilimumab	None	ORR	ORR: 14.3%–33%
SOC-1 (27, 28, 99)	Evaluate secondary cytoreduction in platinum-sensitive relapse	Phase II/III, randomized, open-label	357	Platinum-sensitive OC; TFI >6 months	Secondary cytoreduction	No surgery	OS, PFS	Median PFS: 18.0 vs. 11.9 mo
CARACO (100)	Assess no retroperitoneal lymphadenectomy in primary surgery	Phase III, randomized, open-label	379	Newly diagnosed stage III/IV OC	No retroperitoneal lymphadenectomy	Retroperitoneal lymphadenectomy	PFS	Median PFS: 14.8 vs. 18.5 mo

ADC, antibody–drug conjugate; AE, adverse event; BEV, bevacizumab; CARB, carboplatin; CI, confidence interval; CRR, complete response rate; IDS, interval debulking surgery; HR, hazard ratio; ITT, intention-to-treat; IV, intravenous; mOS, median overall survival; mPFS, median progression-free survival; NR, not reached; OC, OC; ORR, objective response rate; OS, overall survival; PFS, progression-free survival; PLD, pegylated liposomal doxorubicin; TFI, treatment-free interval; TC, paclitaxel and carboplatin; TFI, treatment-free interval.

The DUO-O trial evaluated durvalumab (anti-PD-L1) and olaparib (PARP inhibitor) added to standard chemotherapy plus bevacizumab in newly diagnosed advanced OC. In this phase III study, maintenance with durvalumab + olaparib + bevacizumab significantly prolonged progression-free survival compared with bevacizumab alone (25.1 vs 19.3 months; HR 0.61, 95% CI 0.51–0.73) (90). The benefit was greatest in HRD-positive tumors but extended across biomarker subgroups. DUO-O highlights synergy among PARP inhibition, immune checkpoint blockade, and angiogenesis suppression, underscoring the move toward biomarker-driven combination therapy in frontline OC.

The ATHENA Combo trial assessed the combination of nivolumab (anti-PD-1) and rucaparib (PARP inhibitor) as maintenance therapy following platinum-based chemotherapy in newly diagnosed stage III–IV OC. This phase III, randomized, double-blind study enrolled 863 patients who had responded to first-line platinum treatment. The combination did not improve progression-free survival compared with rucaparib alone (15.0 vs 20.2 months; HR 1.29, 95% CI 1.08–1.53) (91). Although the results were negative, ATHENA Combo provided important insight into the complexity of integrating immune checkpoint blockade with DNA-damage repair inhibition. It underscored that not all combinations achieve additive benefit and highlighted the need for refined patient selection and biomarker-guided approaches to identify responders more precisely.

The NeoPembrOV trial explored neoadjuvant chemoimmunotherapy by combining pembrolizumab (anti–PD-1) with standard platinum-taxane chemotherapy in patients with unresectable stage IIIC/IV high-grade serous or endometrioid OC. This phase II, randomized, open-label study compared pembrolizumab plus chemotherapy with chemotherapy alone, followed by interval debulking surgery and maintenance pembrolizumab. The addition of pembrolizumab was safe and feasible but did not significantly improve progression-free survival (19.4 vs 20.8 months) (93). Despite the absence of clinical benefit in an unselected cohort, NeoPembrOV highlighted the importance of biomarker-guided patient selection and provided valuable evidence supporting the feasibility of introducing immunotherapy earlier in OC treatment.

The ANITA trial evaluated atezolizumab (anti–PD-L1) in combination with platinum-doublet chemotherapy followed by niraparib (PARP inhibitor) maintenance in patients with platinum-sensitive recurrent OC. This phase III, randomized, double-blind study enrolled 417 participants with a treatment-free interval of at least six months. The combination produced a modest improvement in progression-free survival (11.2 vs 10.1 months) compared with the control arm (94). Although the benefit was limited, ANITA confirmed the feasibility of combining immune checkpoint inhibitors with PARP inhibitors and anti-angiogenic agents in the recurrent setting. The trial underscored the importance of long-term safety monitoring and the optimization of combination dosing to balance efficacy and toxicity in multi-agent regimens.

The ATALANTE trial investigated the addition of atezolizumab (anti–PD-L1) to carboplatin-based chemotherapy and bevacizumab maintenance in patients with platinum-sensitive recurrent non-mucinous epithelial OC. This phase III, randomized, double-blind trial included 614 participants with one or two prior treatment lines. The incorporation of atezolizumab resulted in a modest but statistically significant improvement in median progression-free survival (13.6 vs 11.3 months; HR 0.83, *p* = 0.035) (95). Although the clinical benefit was limited, ATALANTE reinforced the rationale for combining immune checkpoint blockade with anti-angiogenic therapy and underscored the need for more precise biomarker-based patient selection to identify subgroups most likely to benefit.

The NRG-GY005 trial examined a non-chemotherapy combination of cediranib (VEGFR inhibitor) and olaparib (PARP inhibitor) versus standard chemotherapy in patients with platinum-resistant or refractory high-grade serous or endometrioid OC. This phase II/III, randomized, open-label study enrolled 562 participants and demonstrated a modest improvement in median progression-free survival (5.2 vs 4.3 months; HR 0.75) compared with chemotherapy (96). While the benefit was limited, the trial provided important validation for chemotherapy-free strategies in heavily pretreated, resistant populations. NRG-GY005 emphasized the growing interest in integrating PARP inhibition with anti-angiogenic and immune-based approaches to expand therapeutic options for patients with few remaining treatment alternatives.

The AGO-OVAR 2.29/ENGOT-ov34 trial evaluated the integration of atezolizumab (anti–PD-L1) with bevacizumab and chemotherapy in patients with recurrent platinum-sensitive high-grade OC. This phase III, randomized, double-blind study enrolled 574 participants to determine whether adding immune checkpoint blockade could enhance clinical outcomes. The inclusion of atezolizumab did not result in a statistically significant improvement in median progression-free survival (6.3 vs 6.6 months; HR 0.88) (97). Despite the lack of clear efficacy, the trial provided valuable data on safety and treatment tolerability, reinforcing the importance of identifying biomarker-defined subgroups that could derive benefit from PD-L1 inhibition in combination with anti-angiogenic regimens.

The LARA trial investigated the combination of pembrolizumab (anti–PD-1) and lenvatinib (tyrosine kinase inhibitor) in patients with recurrent clear cell OC or endometrial carcinoma. This phase II, open-label, two-stage study enrolled 27 patients and reported an ORR of 60% at 24 weeks (98). These findings highlight the therapeutic potential of combining immune checkpoint blockade with anti-angiogenic signaling inhibition in rare, chemoresistant histological subtypes. The trial underscores the growing importance of tailored immunotherapy approaches for molecularly distinct and aggressive OC variants, where conventional platinum-based regimens often yield limited benefit.

## 8 Mechanistic rationale and clinical integration of immunotherapy and ADC-based regimens across gynecologic malignancies

The integration of ICIs, ADCs, and targeted combinations has transformed the treatment paradigm for gynecologic cancers, emphasizing biomarker-guided precision (**Table 7**). In cervical cancer, pembrolizumab in combination with platinum–taxane chemotherapy, with or without bevacizumab, represents a frontline standard for PD-L1–positive disease (KEYNOTE-826 (55)), while tisotumab vedotin (innovaTV 204/301 (54, 55)) has established itself as a second-line ADC option targeting tissue factor (TF). Cemiplimab (EMPOWER-Cervical 1 (53)) further expanded immunotherapy indications to PD-L1–independent settings. Meanwhile, novel investigational combinations such as cadonilimab (dual PD-1/CTLA-4 blockade) and sacituzumab-TMT with pembrolizumab are exploring synergistic immune activation through complementary mechanisms like antigen release and immune cell priming. Collectively, these regimens illustrate how PD-1/PD-L1 blockade and ADC-based cytotoxic delivery are reshaping outcomes in both frontline and refractory disease contexts.

**Table 7:** Mechanistic rationale, predictive biomarkers, and regulatory status of key systemic regimens in cervical, endometrial, and ovarian cancers.

Regimen/Key trial(s)	Biological rationale (proposed synergy)	Predictive biomarkers (if any)	Regulatory status	Guideline endorsement	Indication & line of therapy
Cervical cancer					
Pembrolizumab + chemotherapy ± bevacizumab (KEYNOTE-826 (55))	PD-1 blockade restores cytotoxic T-cell function in HPV-driven tumors; VEGF inhibition (when used) improves immune infiltration and antigen presentation, enhancing ICI activity	PD-L1 CPS ≥ 1 (enrichment); HPV positivity common but not required for selection	FDA/EMA-approved with chemo ± bevacizumab for PD-L1+ disease	NCCN Category 1	First-line, persistent/recurrent/metastatic
Bevacizumab + platinum/taxane (GOG-240 (54))	VEGF blockade reduces angiogenesis and hypoxia, normalizes vasculature, improves drug delivery and antitumor immunity	None required	FDA/EMA-approved with chemo	NCCN Category 1	First-line, persistent/recurrent/metastatic
Cemiplimab (EMPOWER-Cervical 1 (53))	PD-1 blockade reactivates effector T cells after platinum failure	None required; PD-L1 not mandatory	FDA/EMA-approved	NCCN Category 1	Second-line and beyond, post-platinum
Tisotumab vedotin (TV) (innovaTV 204/301 (54, 55))	Anti-TF ADC delivers MMAE to TF-expressing cells; bystander killing and immunogenic cell death possible	TF expression common; no formal companion Dx	FDA/EMA-approved after chemo	NCCN Category 1 (post-chemo)	Second-line and beyond, recurrent/metastatic
Atezolizumab + chemo + bevacizumab (BEATcc (51))	PD-L1 blockade plus VEGF inhibition (normalizes microenvironment) and cytotoxic chemo → complementary immune and anti-angiogenic effects	None mandated; activity seen irrespective of PD-L1	Investigational (positive phase III)	Not yet in NCCN as standard	First-line, persistent/recurrent/metastatic
Cadonilimab (PD-1/CTLA-4) + chemo ± bevacizumab (COMPASSION-16 (53))	Dual checkpoint blockade enhances T-cell priming and effector phases; possible benefit even when PD-L1 negative	Exploratory PD-L1; no required Dx	Investigational	Not in NCCN	First-line, persistent/recurrent/metastatic
Sacituzumab-TMT + pembrolizumab (SKB264-II-06 (52))	Trop-2 ADC induces tumor cell death and antigen release; PD-1 blockade amplifies immune recognition (immunogenic cell death synergy)	Trop-2 expression frequent; exploratory	Investigational (phase II)	Not in NCCN	Post-platinum setting
Endometrial cancer					
Pembrolizumab + carboplatin/paclitaxel (NRG-GY018 (82))	PD-1 blockade restores effector T-cell activity; cytotoxic chemotherapy promotes antigen release and immune priming, creating synergistic tumor clearance	dMMR/MSI-H (strong), PD-L1 (supportive), TMB-high	FDA/EMA-approved 2024 for advanced/recurrent dMMR/MSI-H EC	NCCN Category 1	1st-line, advanced/recurrent EC
Dostarlimab + carboplatin/paclitaxel (RUBY Part 1 (85))	PD-1 inhibition augments immune recognition of dMMR tumors and complements chemotherapy-induced immunogenic cell death	dMMR/MSI-H	FDA-approved 2023 for dMMR advanced/recurrent EC	NCCN Category 1	1st-line, advanced/recurrent EC
Pembrolizumab + lenvatinib (KEYNOTE-775/LEAP-001 (83))	VEGFR/FGFR/PDGFR inhibition by lenvatinib remodels vasculature and reduces immunosuppressive cytokines, sensitizing pMMR tumors to PD-1 blockade	pMMR (benefit predominant), PD-L1 variable	FDA/EMA-approved 2021 for pMMR advanced EC after platinum	NCCN Category 1	2nd-line, pMMR advanced/recurrent
Durvalumab ± olaparib + chemo (DUO-E/ENGOT-en9/DUO-O (86))	Synergy through immune checkpoint inhibition and PARP-mediated DNA-damage accumulation; enhances neoantigen load and immune activation	HRD/BRCA mutations, dMMR/MSI-H	Investigational, positive phase III	Pending in NCCN	1st-line and maintenance (under evaluation)
Atezolizumab + carboplatin/paclitaxel (AtTEnd (87))	PD-L1 blockade re-activates antitumor immunity; chemotherapy provides immunogenic stimulus	PD-L1 expression, dMMR subgroup favorable	Investigational (phase III)	Not yet in NCCN	1st-line, advanced/recurrent
Sacituzumab govitecan (TOTEM (88, 89))	Trop-2 ADC delivers SN-38 payload causing DNA damage and bystander tumor killing; may generate secondary immune activation	Trop-2 expression (common in EC)	Investigational, early phase II	Not in NCCN	Heavily pretreated/recurrent
Ovarian cancer					
Durvalumab + olaparib + bevacizumab after chemo-bev (DUO-O) (90)	PARP inhibition increases DNA damage and neoantigen load, VEGF blockade normalizes vasculature and reduces immunosuppression, PD-L1 blockade restores T-cell function → triplet synergy	HRD, BRCA status, HRD-associated signatures	Investigational in OC	Not standard in NCCN/ESMO for OC	Frontline stage III–IV, maintenance after chemo-bev
Nivolumab + rucaparib maintenance (ATHENA Combo) (91, 92)	PARP-induced immunogenicity plus PD-1 blockade	HRD, BRCA, tumor immune infiltration	Investigational; negative signal	Not endorsed	Frontline maintenance post-platinum
Pembrolizumab added to neoadjuvant chemo, then maintenance (NeoPembrOV) (93)	Chemo promotes antigen release and dendritic priming, PD-1 blockade augments effector function	PD-L1 CPS, TMB, immune gene signatures	Investigational	Not endorsed	Neoadjuvant in unresectable stage IIIC/IV
Atezolizumab + platinum doublet, niraparib maintenance (ANITA) (94)	PD-L1 blockade plus PARP-induced DNA damage; potential chemo-immunogenic synergy	HRD, BRCA, PD-L1	Investigational	Not endorsed	Platinum-sensitive recurrence
Atezolizumab + carboplatin-based chemo + bev maintenance (ATALANTE) (95)	PD-L1 blockade with anti-angiogenic normalization and chemo-induced immunogenic death	PD-L1, immune infiltration	Investigational	Not endorsed	Platinum-sensitive recurrent non-mucinous
Cediranib + olaparib vs chemo (NRG-GY005) (96)	VEGFR blockade remodels TME and augments PARP effect; chemo-free strategy	HRD, BRCA, angiogenic signatures	Investigational	Not endorsed	Platinum-resistant or refractory
Atezolizumab + bevacizumab + chemo (AGO-OVAR 2.29/ENGOT-ov34) (97)	PD-L1 blockade plus VEGF inhibition and chemo	PD-L1, immune signatures	Investigational; no benefit	Not endorsed	Platinum-sensitive recurrence
Pembrolizumab + lenvatinib in clear cell OC (LARA) (98)	Multi-TKI anti-angiogenic and immunomodulatory effects sensitize to PD-1 blockade; clear cell may be immune-inflamed	Clear cell histology, PD-L1	Investigational in OC	Not endorsed	Recurrent clear cell OC (small phase II)
Nivolumab ± ipilimumab in clear cell OC (BrUOG 354) (99)	Dual checkpoint blockade enhances T-cell priming and effector function	Clear cell histology, PD-L1, TMB	Investigational	Not endorsed	Recurrent clear cell OC

In endometrial and ovarian cancers, ongoing advances underscore the growing role of multi-pathway synergy. In endometrial carcinoma, PD-1 inhibitors combined with chemotherapy or anti-angiogenic agents have achieved FDA/EMA approval across molecular subtypes, including pembrolizumab plus carboplatin/paclitaxel (NRG-GY018 (82)), dostarlimab plus chemotherapy (RUBY Part 1 (85)), and pembrolizumab plus lenvatinib (KEYNOTE-775 (83)), now standard for pMMR disease. The addition of durvalumab with PARP inhibition (DUO-E (86)) and early ADC strategies (TOTEM (88, 89)) reflect future directions toward integrated, biomarker-driven regimens. In ovarian cancer, immunotherapy remains investigational but promising, with triplet combinations like durvalumab + olaparib + bevacizumab (DUO-O (90)) showing synergistic potential through DNA-damage accumulation and immune activation. Although other trials (ATHENA Combo, ANITA, ATALANTE) yielded modest or negative results, exploratory regimens such as pembrolizumab + lenvatinib and nivolumab ± ipilimumab in clear cell subtypes highlight the ongoing refinement of immunologic and molecularly tailored strategies. Together, these findings consolidate a unified framework for precision immunotherapy across gynecologic malignancies, balancing established standards with emerging targeted innovations.

In ovarian cancer, the therapeutic landscape is undergoing a steady evolution toward rationally designed combinations that leverage DNA repair targeting, angiogenesis modulation, and immune activation. While immune checkpoint inhibitors alone have shown limited efficacy in unselected populations, combining them with PARP inhibitors and anti-angiogenic agents has yielded promising signals in biomarker-defined subgroups. The DUO-O trial (90) exemplifies this progress, demonstrating that the addition of durvalumab and olaparib to standard bevacizumab maintenance significantly prolonged progression-free survival, particularly in HRD-positive tumors. This triplet approach capitalizes on complementary mechanisms: PARP inhibition induces DNA damage and neoantigen release, VEGF blockade improves immune infiltration, and PD-L1 inhibition reactivates exhausted T cells. Other investigations, such as ANITA (94) and ATALANTE (95), have validated the biological rationale for integrating PD-L1 blockade with PARP inhibitors and anti-angiogenic therapy, though their clinical gains were modest. Novel chemo-free strategies, including cediranib plus olaparib (NRG-GY005 (96)), further illustrate the pursuit of durable, less toxic regimens for platinum-resistant disease. Importantly, rare and chemoresistant histotypes like clear cell ovarian carcinoma are emerging as distinct immunogenic subgroups responsive to dual checkpoint blockade or PD-1–TKI combinations, as shown in LARA (98) and BrUOG 354 (99). Overall, these findings underscore a shift from conventional cytotoxic therapy toward integrated, molecularly informed regimens that aim to achieve sustained disease control through multi-target immune modulation.

## 9 Integration of artificial intelligence, genomic profiling, and biomarker-driven strategies in gynecologic oncology

Artificial intelligence (AI) is increasingly being applied across gynecologic oncology to enhance precision medicine by integrating genomic, proteomic, and clinical data (102). Early frameworks such as the MIA3G and MCF models demonstrated how multi-parameter learning systems can stratify OC risk and improve diagnostic accuracy using combinations of biomarkers like CA125, HE4, and β_2_-microglobulin (103, 104). These approaches achieved high sensitivity and specificity in retrospective datasets and highlighted the capacity of AI to extract non-linear diagnostic patterns beyond traditional statistical models (105–107). Despite these advances, interpretability and limited external validation remain challenges, emphasizing the need for explainable AI (XAI) and prospective, multi-institutional verification (**Table 8**) (108–114).

**Table 8:** AI in gynecologic oncology surgery.

Cancer type	AI model	Purpose	Input variables	Outcome measures	Clinical impact	Limitations/Notes	Ref.
Other	XGBoost, RF, LR	Residual disease prediction after hysterectomy	Clinical and surgical parameters	Identification of top predictors (e.g., diaphragm, bowel mesentery involvement)	Improved adjuvant treatment planning	Models similar in performance	(108)
Other	Custom predictive model	Postoperative morbidity and mortality risk	GO SOAR database parameters	30-day morbidity and mortality prediction	Development of surgical risk calculator	Validated with preliminary data	(109, 110)
OC	ML algorithms	Predict resectability in HGSOC	Clinical and imaging data	Identified pelvic and intestinal carcinosis as non-resectable criteria	Reduced ‘open & close’ surgeries	Need prospective validation	(111)
OC	ML/DL	Length of stay prediction after cytoreductive surgery	Age, BMI, ECOG PS, time, SCS, blood loss	LOS prediction via Leeds L-AI-OS Score	Enhanced surgical planning	May need external validation	(112)
OC	GUI Calculator	ICU admission risk	Same as above + ostomy	Natal Score developed	Improved ICU resource planning	Needs broader testing	(113)
OC	XGBoost, DNN	Cytoreduction success and prognosis	Surgical features (e.g., bowel, diaphragm)	ANAFI score and SCS cut-off developed	Intraoperative guidance	Focus on anatomical patterns	(114–116)
OC	Unspecified	Prediction of complete cytoreduction	Surgical procedures	UAP and lymphadenectomy as predictors	Model for surgical planning	Used ESGO-accredited data	(117)
OC	Biomarker analysis	Predict surgical outcome	HE4 and CA125	Prediction of suboptimal cytoreduction	Risk stratification	Needs further validation	(118)
OC	ANN	Predict success of secondary cytoreduction	DFI, recurrence site, FIGO stage	Complete resection and OS	Surgical candidate selection	Retrospective, limited sample	(119)
Endometrial cancer	Various ML models	Predict EC with EIN	Histologic and clinical data	Sensitivity <50%	Currently not predictive	Low model accuracy	(120, 121)
Endometrial cancer	Metabolomics + ML	Non-invasive EC screening	Blood metabolites	Metabolites identified	Early detection, personalized therapy	Needs standardization	(122)
Cervical cancer	iPMI	Predict parametrial invasion	Imaging and clinical features	Cost-effective alternative	Guided surgical planning	In early development	(123)
Cervical cancer	Deep Learning	Predict survival	Postoperative variables	Survival prediction	Outcome stratification	Preliminary results	(114)

AI methodologies have also been introduced into surgical oncology for risk assessment and perioperative optimization. Machine-learning models such as XGBoost, random-forest, and deep-learning frameworks predict cytoreductive outcomes, postoperative morbidity, and intensive-care needs by analyzing clinical and imaging data (108–116). These models identify key variables including diaphragm and bowel involvement or estimated blood loss, which aid intraoperative decision-making and improve surgical planning (117–119). Comparable tools have been developed for endometrial and cervical cancers, integrating metabolomic or radiologic data to predict parametrial invasion and survival outcomes (120–123). Collectively, these algorithms exemplify AI’s capacity to personalize surgical strategies and reduce complications.

Recent investigations have broadened AI’s role in molecular prediction. Models integrating circulating-free DNA (cfDNA), metabolomic profiles, and multi-omics features enhance early detection of OC and uterine corpus endometrial carcinoma (UCEC) (104, 124–135). Frameworks such as DELFI, based on cfDNA fragmentomics, and GC–MS-based metabolomic classifiers demonstrated robust AUC values and accuracy exceeding 90%, underscoring AI’s diagnostic strength for minimally invasive screening (128–133). However, dataset heterogeneity, population bias, and lack of standardized analytic pipelines still constrain clinical translation.

**Figure 3:**
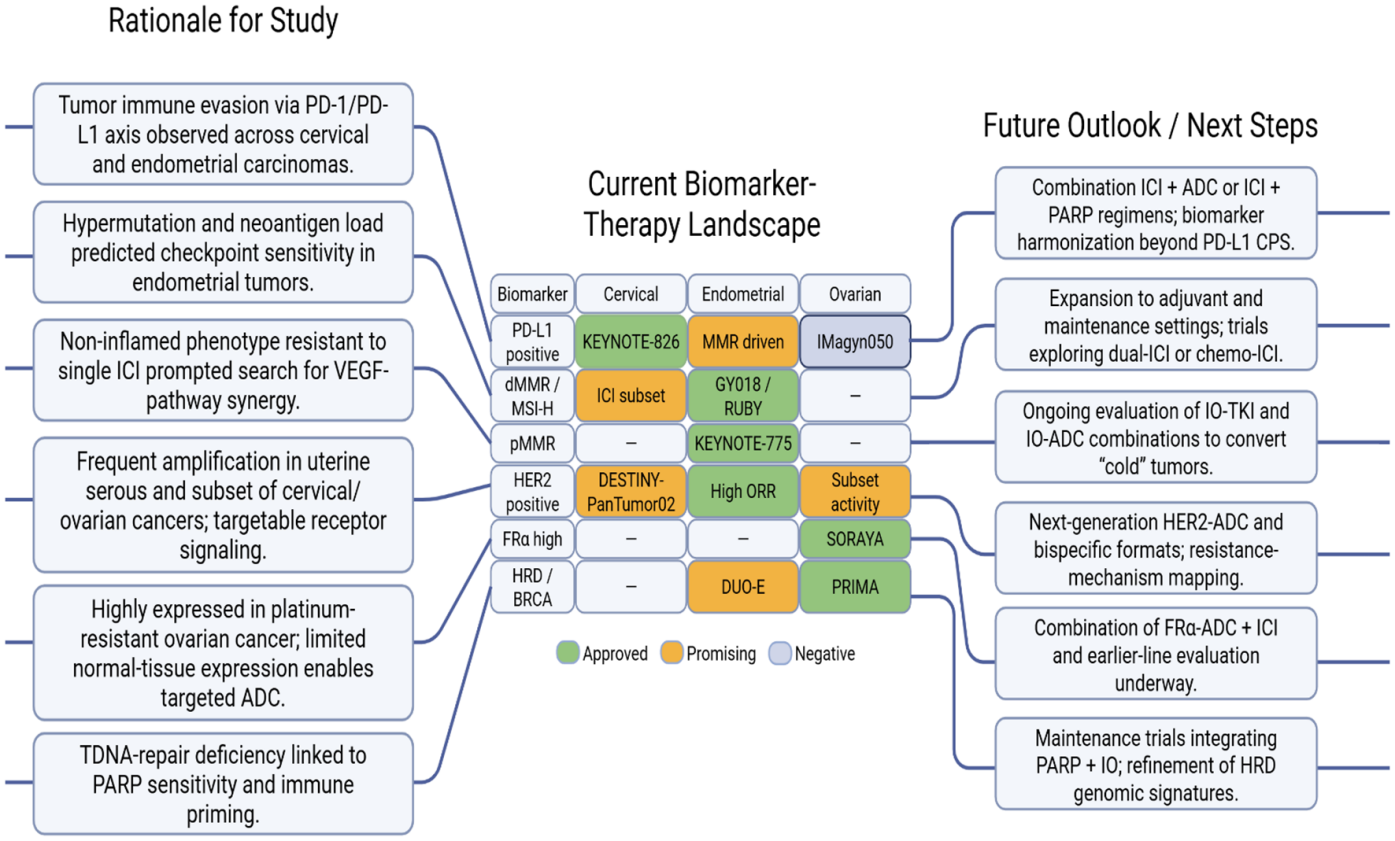
Translational map of immunotherapy and ADC deployment across gynecologic cancers.

Beyond diagnostics and surgical optimization, artificial intelligence can play a transformative role in integrating immunotherapy and ADCs into the therapeutic continuum of gynecologic malignancies (136). Machine learning models could identify predictive immune signatures such as PD-L1 expression patterns, tumor mutational burden, microsatellite instability, and immune cell infiltration to guide patient selection for checkpoint blockade therapies. Similarly, AI-driven multi-omics integration could uncover molecular determinants of ADC response, including HER2 expression, receptor density, and intracellular trafficking efficiency, thereby improving target validation and therapeutic precision (137). Advanced predictive frameworks may also enable dynamic treatment adaptation by correlating radiomic or liquid biopsy data with immune response kinetics, helping clinicians anticipate resistance or toxicity in real time. In the long term, explainable AI systems combining genomic, proteomic, and histopathologic data could serve as clinical decision-support tools, harmonizing the use of immunotherapy and ADCs within personalized gynecologic oncology treatment algorithms (**Figure 3**). While the reviewed studies demonstrate considerable promise, significant challenges endure. Numerous investigations rely on retrospective data or narrowly defined cohorts, which may restrict external validity. Additionally, the regulatory frameworks for AI-driven diagnostics are still in nascent stages, necessitating the establishment of harmonized standards for validation, transparency, and clinical incorporation. Lastly, the interpretability of AI models is a pivotal concern in clinical environments, where explainable AI (XAI) could significantly facilitate clinician acceptance (136).

Future investigations must prioritize prospective, multicenter validation, encompass diverse ethnic groups, and include cost-effectiveness evaluations to guarantee equitable implementation. Furthermore, the synergy with multi-omics data (genomics, transcriptomics, metabolomics, and proteomics) could lead to the development of highly personalized and robust diagnostic instruments that surpass existing biomarker constraints.

## 10 Conclusions

Over the past decade, treatment strategies for gynecologic malignancies including cervical, endometrial, and ovarian cancers have shifted toward biomarker-driven and immune-modulated approaches. Conventional cytotoxic chemotherapy and surgery remain the cornerstone of care, yet targeted agents, ADCs, and ICIs are now integral to modern management. Among the established standards of care, pembrolizumab combined with platinum-based chemotherapy for PD-L1-positive cervical cancer (KEYNOTE-826), dostarlimab with chemotherapy for dMMR endometrial cancer (RUBY Part 1), and PARP inhibitor maintenance for HRD-positive ovarian cancer (PRIMA, DUO-O) are incorporated into international guidelines and have received FDA or EMA approval. These regimens have redefined first-line management through molecular selection and durable survival benefit.

Promising but still investigational options include multi-agent combinations such as durvalumab with olaparib (DUO-E), atezolizumab-based chemoimmunotherapy (ATALANTE, ANITA), and next-generation ADCs including trastuzumab deruxtecan and sacituzumab govitecan. These regimens show encouraging activity but require extended follow-up and biomarker validation before full clinical endorsement. Artificial-intelligence-assisted clinical decision models and surgical outcome prediction tools also belong to this emerging category. Although their early performance is strong, broader validation is needed before routine use in perioperative care.

Approaches that currently lack clear clinical benefit include PARP inhibitor–immunotherapy combinations without biomarker selection such as ATHENA Combo and NeoPembrOV. Their limited synergy illustrates the importance of rational trial design based on immune profiling, DNA repair status, and tumor microenvironment biology rather than empirical combination.

Future work should focus on refining predictive biomarkers such as HRD, MSI-H/dMMR, PD-L1, and HER2 expression and integrating them into clinical algorithms. The use of genomic, proteomic, and radiomic data analyzed through transparent AI models may help optimize patient selection and anticipate resistance. Equal attention should be given to cost-effectiveness and access to high-value therapies in resource-limited regions.

In summary, gynecologic oncology is rapidly moving from empiric therapy toward precision-based treatment. Immunotherapy and ADCs now represent standards for biomarker-defined patient groups, while combination strategies and AI-assisted approaches continue to evolve. Progress will depend on multidisciplinary collaboration, innovative trial design, and harmonized biomarker testing to ensure that advances in precision medicine translate into better survival and quality of life for women worldwide.
