# Correction: Recent therapeutic advances in gynecologic oncology: evolving roles of immunotherapy, antibody–drug conjugates, and clinical trial innovations

**DOI:** 10.3389/fonc.2026.1803643

**Published:** 2026-03-24

**Authors:** Gaukhar Koshkimbayeva, Akerke Amirkhanova, Aiken Orazymbetova, Alma Nurakhova, Akmaral Maimakova, Altyn Duisenbayeva, Nurgulim Akhmad, Altyn Abilova, Arailym Abilbayeva, Sholpan Akhelova, Dana Akhmentayeva, Aida Seitaliyeva, Zaure Dushimova, Zhanserik Shynykul, Sandugash Yerkenova

**Affiliations:** 1 Department of General Medical Practice with Courses, Kazakh-Russian Medical University, Almaty, Kazakhstan; 2 School of Pharmacy, S.D. Asfendiyarov Kazakh National Medical University, Almaty, Kazakhstan; 3 Department of Anatomy with Courses, Kazakh-Russian Medical University, Almaty, Kazakhstan; 4 Department of Normal Physiology with a course in Biophysics, School of Medicine, S.D. Asfendiyarov Kazakh National Medical University, Almaty, Kazakhstan; 5 Clinical Medicine Department of International Business, University named after Kenzhegali Sagadiev, Almaty, Kazakhstan; 6 Department of Anatomy named after S. R. Karynbayev, School of General Medicine 1, S.D. Asfendiyarov Kazakh National Medical University, Almaty, Kazakhstan; 7 General Immunology Department, S.D. Asfendiyarov Kazakh National Medical University, Almaty, Kazakhstan; 8 General Immunology Department, S.D. Asfendiyarov Kazakh National Medical University, Almaty, Kazakhstan; 9 Department of Pharmaceutical Disciplines, NCJSC “Astana Medical University”, Astana, Kazakhstan; 10 Department of Internal Medicine, S.D. Asfendiyarov Kazakh National Medical University, Almaty, Kazakhstan; 11 Faculty of Medicine and Healthcare, Al-Farabi Kazakh National University, Almaty, Kazakhstan; 12 Department of Anatomy, Kazakhstan’s Medical University “KSPH”, Almaty, Kazakhstan

**Keywords:** gynecologic malignancies, immune checkpoint inhibitors, antibody–drug conjugates, ovarian cancer, endometrial cancer, cervical cancer, cancer

Affiliation “Department of Anatomy named after S. R. Karynbayev, School of General Medicine 1, S.D. Asfendiyarov Kazakh National Medical University, Almaty, Kazakhstan” was erroneously given as an incorrect affiliation for “Nurgulim Akhmad and Altyn Abilova”.

Affiliation “Department of Anatomy with Courses, Kazakh-Russian Medical University, Almaty, Republic of Kazakhstan” was erroneously given as an incorrect affiliation for “Aiken Orazymbetova”.

The original version of this article has been updated.

